# A Comparative Study of Virucidal and Virustatic Multivalent Entry Inhibitors

**DOI:** 10.1021/acs.jpcb.5c05864

**Published:** 2025-10-28

**Authors:** Hien Thi Tran, Sujeet Pawar, Yong Zhu, Quy Khac Ong, Francesco Stellacci

**Affiliations:** † Institute of Materials, 27218 École Polytechnique Fédérale de Lausanne, Station 12, CH-1015 Lausanne, Switzerland; ‡ Institute of Bioengineering, École Polytechnique Fédérale de Lausanne (EPFL), Station 12, CH-1015 Lausanne, Switzerland; § Global Health Institute, École Polytechnique Fédérale de Lausanne (EPFL), Station 12, CH-1015 Lausanne, Switzerland

## Abstract

Viral infections, such as those caused by herpes simplex viruses (HSV) and influenza, continue to pose a significant global health challenge. We have focused on the development of multivalent entry inhibitors (MEIs) that have an irreversible inhibition mechanism, i.e., virucidal, as opposed to the commonly found reversible virustatic ones. MEIs are typically composed of core structures connected to multiple functional groups that are engineered to bind to viruses. In between the core and the functional groups, we inserted alkyl linkers and showed that such linkers, when long enough, were responsible for a change in the inhibition mechanism by their hydrophobicity. In a recent paper, we found that comparison of the antiviral properties against HSV-2 of one pair of sulfonate and sulfate MEIs had led to a surprising result. The compounds shared the same core (benzene) and had three undecyl linkers that were terminated either by sodium sulfates or by sodium sulfonates, respectively. The former showed a virucidal and the latter a virustatic inhibition mechanism. In this paper, we show that such a surprising difference is also true when testing these compounds against HSV-1 and a few different influenza strains. This difference remains when the hydrophobic linkers are shorter (hexyl). For these four MEIs, we present a series of measurements aimed at determining the hydrophobicity (critical micelle concentration [CMC] and partition coefficient [Log*P*]) and their binding with proteins. We find that the only parameters that correlate positively with the virucidal mechanism are the interactions of the compounds with bovine serum albumin and Log*P*. We interpret our data as indicating that what matters for a virucidal mechanism is the ability of a MEI to establish hydrophobic interactions with proteins in solution.

## Graphical abstract

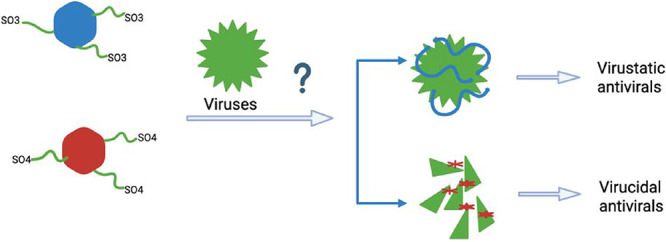

## Introduction

Emerging viruses pose a significant public health threat, with three to four new species identified each year.^1^ These viruses are associated with a wide range of diseases, including influenza, which accounts for 3–5 million cases of severe illness annually.^2^ Herpes simplex virus type 2 (HSV-2), for example, was estimated to affect 491.5 million people globally in 2016, representing 13.2% of the population aged 15–49 years.^3^ Similarly, herpes simplex virus type 1 (HSV-1) was reported to infect approximately 3.7 billion individuals in the same year, accounting for 66.6% of the population aged 0–49 years. To prevent and treat viral diseases, two primary approaches exist: vaccines and antiviral compounds. Viral vaccines are typically developed using live-attenuated or inactivated viruses, viral surface proteins, neutralizing antibodies, or nucleic acids capable of producing surface proteins.^4–6^ However, it has been recognized that developing effective vaccines remains highly challenging for viruses characterized by chronic infection, immune evasion, viral genome integration, or delayed activation of adaptive immunity.^7^ As early as the 1960s, many scientists doubted whether it was possible to create drugs that could specifically target viruses. At present, 106 antiviral drugs are approved and used to treat viral diseases. These drugs have saved tens of millions of lives and continue to play a key role in fighting current and emerging viral infections.

Based on their mechanism of action, antivirals can be separated into intracellular and extracellular. The intracellular ones are typically enzyme inhibitors, while the extracellular ones are entry inhibitors. Entry inhibitors can be further grouped into two major classes: virucidal and virustatic. Virustatic drugs are known for their ability to hinder viral infection through a reversible extracellular mechanism.^8^ Upon dilution in bodily fluids below a certain binding threshold, these drugs are released from the virus, allowing the intact virus to regain infectivity. In contrast, virucidal drugs act on viruses through an irreversible extracellular mechanism.^9^ These agents primarily work by damaging the virion’s protein capsid or outer membrane, or by penetrating the virion to damage its genome, thereby compromising the integrity of the viral particle.^10^ While both virustatic and virucidal drugs possess many desirable attributes, virustatic compounds often lack efficacy in vivo, whereas virucidal drugs, in most cases, are excessively toxic.

Developing antiviral drugs that can work against multiple types of viruses is especially important for coping with current and emerging viruses. A defining feature of current broad-spectrum antivirals is their extracellular mode of action.^10^ Following this reason, the interaction between viral proteins and surface glycan of host cells is a common occurrence in many viral infections. Heparan sulfate, a sulfated glycosaminoglycan, and sialic acid are common sugars that frequently act as coreceptors, facilitating the attachment of viral particles to host cells before entry.^11^ One approach to achieving this is by mimicking cell surface receptors like heparan sulfate proteoglycans (HSPGs) or sialic acids.^12,13^ Both natural and synthetic sulfated or sulfonated materials have long been recognized for their ability to function as HSPG mimics, binding to viruses and exhibiting antiviral properties.^14,15^ Recently, various (supra)­molecular structures have been proposed as broad-spectrum sulfate or sulfonate MEIs, such as nanoparticles, cyclodextrins, dendritic polyglycerols (dPG), lipid-interacting agents, polyanionic compounds, peptide-based virucidals, and surfactants.^16–22^ Some of us reported a broad-spectrum antiviral based on gold nanoparticles (AuNPs).^16^ We showed that we could modify known virustatic AuNPs into virucidal AuNPs by replacing short 2-mercaptoethanesulfonic acid (MES) ligands with the 11-mercapto-1-undecanesulfonic acid (MUS) ligands.^17^ The particles were shown to inhibit various viruses and disrupt their structures. Later, we developed a similar virucidal MEI by replacing the AuNP core with a cyclodextrin (CD). CD-MUS exhibited micromolar virucidal broad-spectrum inhibition.^17^ The role of linker length and structure was examined. It was found that shortening the linker to seven carbon atoms eliminated virus inhibition, while using a rigid linker with aromatic rings reduced virucidal activity. To enhance multivalency and improve antiviral potency, we synthesized a series of dPG modified with hydrophobic sulfonated or sulfated ligands.^18^ We showed that the length of the alkyl chains influences the antiviral properties: longer chains promote virucidal effects, while shorter chains lead to virustatic ones. Although many papers have investigated sulfate- or sulfonate-based antivirals, most focus on the synthesis and inhibition mechanisms of individual compounds. However, no specific studies have directly compared their virucidal and virustatic properties in relation to functional groups, such as sulfate and sulfonate.

Despite many indications on the role of hydrophobic linkers in imparting virucidal properties to MEI, one must notice that most studies were done on MEIs that did not have a well-defined stoichiometry, thus impairing rigorous comparison of their inhibition mechanism and efficacy. Recently, we developed a series of chemically defined small-molecule MEIs based on a benzene core.^22^ These benzene derivatives with varying alkyl linkers demonstrate significant differences in antiviral efficacy depending on the length and hydrophobicity of these linkers. For example, the IC_50_ (concentration at half maximal inhibitory) value of a B3C11SO4 compound (B-benzene, 3 = three linkers, C11 = an 11-methylenes long linker, SO4 = sulfate) was found to be 26.2 μM against HSV-2, but reducing the length of the linker from 11 to 6 in compound B3C6SO4 increased the IC_50_ value to 78.4 μM.^22^ Unusually, we noted that two benzene derivatives (B3C11SO4 and B3C11SO3) exhibit differences in inhibition mechanisms: B3C11SO4 is virucidal, while B3C11SO3 is virustatic with HSV-2.^22^

In this study, we conducted an in-depth investigation into the differences in virucidal and virustatic properties by comparing sulfate and sulfonate pairs, aiming to identify the molecular characteristics that are essential for achieving virucidal inhibition. We assessed the MEI's ability to bind to viral proteins, as well as to a model protein (BSA-bovine serum albumin) and their partition coefficient, critical micelle concentration (CMC). These experiments showed that the hydrophobic interaction plays a significant role in the virucidal mechanism. Consequently, we evaluated the differences in inhibition properties in pretreatment and posttreatment conditions. The results indicated that virucidal compounds prioritize virus inhibition in the pretreatment condition, while virustatic compounds favor virus inhibition in the posttreatment condition.

## Materials and Methods

Antiviral compounds in this study were synthesized following our published procedures.^22,23^ The details of chemical synthesis and characterization were presented in Supporting Materials (section 2), and the MS and NMR results are presented from Figures SI17–SI31.

### Materials

Dulbecco’s Modified Eagle’s Medium (DMEM) with GlutaMAX, fetal bovine serum (FBS), and 1% penicillin/streptavidin (P/S), PBS 1× were purchased from Life Technologies, USA. Methylcellulose, crystal violet, and BSA were purchased from Sigma-Aldrich, USA. Recombinant glycoprotein D was purchased from Labforce AG, and recombinant H1N1 (A/California/04/2009) hemagglutinin (HA1) was obtained from Sino Biological Inc.

Vero cells (African green monkey kidney epithelial cells) were purchased from ATCC, USA. HSV-2 (herpes simplex virus type 2) and HSV-1 (herpes simplex virus type 1) were kindly provided by Dr. Remi La Polla (EPFL, Switzerland). They were propagated on Vero cells. Four influenza viruses were used in the study: A/Netherlands/602/2009 (H1N1), H1N1 A/Lausanne/2022 clinical, H3N2 A/Wyoming/2003/3, and B/Washington/02/2019 were provided by Dr. Valeria Cagno from Lausanne University Hospital.

### Methods

#### Synthesis of B3C11SO4 ^22^

The synthesis of B3C11OH is presented in Scheme 1. A 100 mL flask was charged with 11-mercapto-1-undecanol (1.83 g, 8.95 mmol, 3.15 equiv), K_2_CO_3_ (1.237 g, 8.95 mmol), and dry ethanol (40 mL). The mixture was stirred for 15 min, and 1,3,5-tris­(bromomethyl)­benzene (1 g, 2.83 mmol) was added. The white suspension was refluxed at 80 °C overnight, cooled down to room temperature, and the solvent removed under reduced pressure to dryness. The white solid was resuspended in DCM (50 mL at reflux), filtered, and washed with DCM (2 × 50 mL). After removal of DCM under vacuo, the residue was recrystallized in ethanol. The white solid was isolated from the mother liquor by filtration, washed with acetonitrile (2 × 20 mL), then diethyl ether (20 mL), and finally dried under vacuum to afford the product (yield = 80%).

**Scheme 1:**
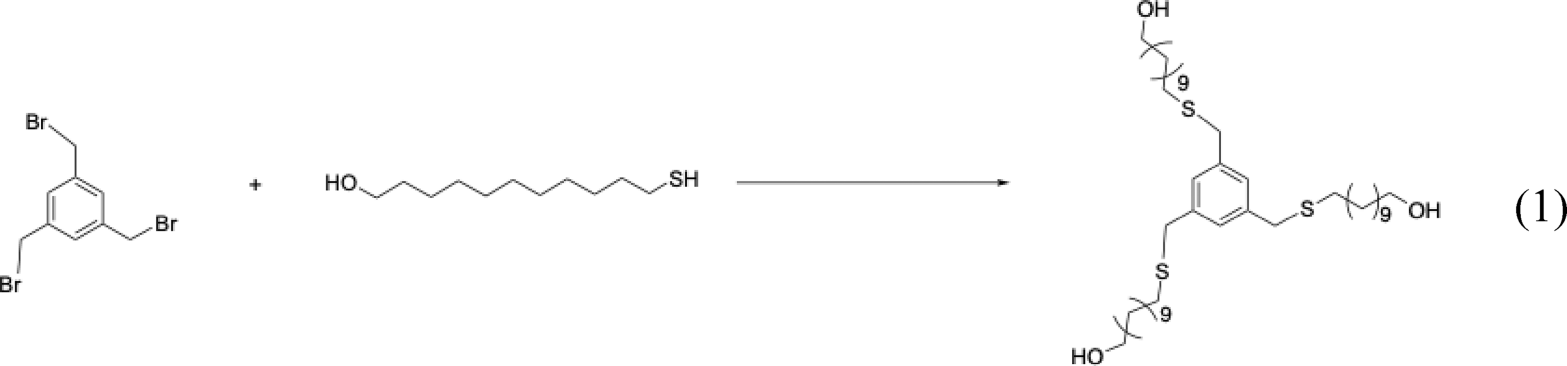
Synthesis of B3C11OH

TLC was used to check the purity of the product. The *R*_f_ value of the product is around 0.3 at the normal phase TLC plate (with developing solvent DCM/MeOH = 20/1 and H_2_SO_4_ stain).

##### The Characterizations of the Product Include NMR and MS

^1^H NMR (400 MHz, CDCl_3_) δ 7.11 (s, 3H, Ar–H), 3.65 (s, 6H, Ar–CH_2_), 3.61 (t, *J* = 6.6 Hz, 6H, CH_2_–O), 2.37 (t, *J* = 7.4 Hz, 6H), 1.74 (s, 4H), 1.62 – 1.15 (m, 60H). ^13^C NMR (101 MHz, CDCl_3_) δ: 139.04, 127.93, 62.98, 36.08, 32.78, 31.43, 29.60, 29.54, 29.53, 29.44, 29.27, 29.25, 28.93, 25.76. HRMS (nanochip-ESI/LTQ-Orbitrap) *m*/*z*: [M + Na]^+^ Calcd for C_42_H_78_NaO_3_S_3_^+^ 749.5005; Found 749.5024.

From compound B3C11OH, following the general method B, gave compound B3C11SO4 as a white powder (yield = 61.8%).

The synthesis of B3C11SO4 is presented in Scheme 2. A two-neck round-bottom flask was charged with B3C11OH (41 mg) and pyridine sulfur trioxide complex (275 mg) under argon. Anhydrous DMF (50 mL) was added, and the reaction mixture was heated at 60 °C for 16 h. After cooling with an ice bath, tributylamine (410 μL) was added, and the reaction mixture was stirred for 30 min at RT. Sodium 2-ethylhexanoate (291 mg, 16 mmol) was added, and the reaction mixture was stirred vigorously for 30 min at room temperature. The volatiles were removed under reduced pressure, EtOH (30 mL) was added, and the mixture was stirred for 30 min. The precipitate was isolated by centrifugation, washed thoroughly with EtOH, and finally dried under a vacuum to obtain the product.

**Scheme 2:**
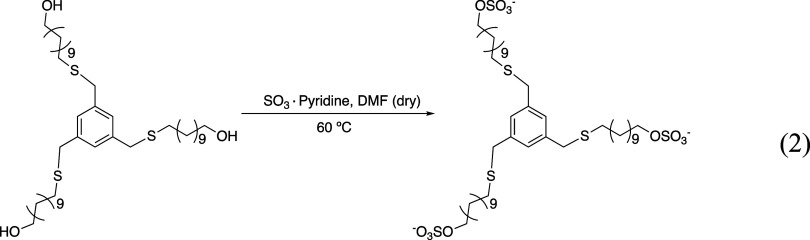
Synthesis of B3C11SO4

##### The Characterizations of the Product Include NMR and MS

^1^H NMR (400 MHz, D_2_O) δ 7.13 (s, 3H, Ar–H), 4.03 (t, *J* = 6.7 Hz, 6H, CH_2_–SO_4_−), 3.64 (s, 6H, Ar–CH_2_), 2.37 (d, *J* = 7.6 Hz, 6H, S–CH_2_), 1.80–1.09 (m, 54H, CH_2_–CH_2_–CH_2_). ^13^C NMR (101 MHz, D_2_O) δ: 139.50, 128.33, 69.68, 36.51, 31.85, 30.12, 29.97, 29.95, 29.83, 29.70, 29.48, 25.92. HRMS (nanochip-ESI/LTQ-Orbitrap) *m*/*z*: [M]^3–^ Calcd for C_42_H_75_O_12_S_6_^3–^ 321.1200; Found 321.12007.

#### Synthesis of B3C11SO3 ^22^

The synthesis of B3C11SO3 is presented in Scheme 3. NaH (60% dispersed in mineral oil, 168 mg, 4.2 mmol) was added to a solution of sodium 11-mercaptoundecane-1-sulfonate (1.22 g, 4.2 mmol) in dry DMF (15 mL). After 30 min of stirring at R.T., a solution of 1,3,5-tris­(bromomethyl)­benzene (357 mg, 1 mmol) in dry DMF (10 mL) was added. The temperature was raised to 60 °C, and the stirring continued overnight. After cooling to rt, ethanol (20 mL) was added, and the solvent was removed under vacuum. The solid residue was purified by reversed-phase flash chromatography (C18, ACN/water gradient) to afford the product (yield = 81%) as a white solid. ^1^H NMR (400 MHz, D_2_O) δ 7.13 (s, 3H, Ar–H), 3.64 (s, 6H, Ar–CH_2_), 2.86–2.9 (t, *J* = 6.7 Hz, 6H, CH_2_–SO_4_−), 2.37 (d, *J* = 7.6 Hz, 6H, S–CH_2_), 1.91–1.29 (m, 42H, CH_2_–CH_2_–CH_2_).^13^C NMR (101 MHz, DMSO-*d*_6_) δ 139.33, 128.18, 51.98, 35.32, 30.90, 29.51, 29.47, 29.45, 29.26, 29.12, 28.96, 28.80, 25.57. HR-MS (nanochip-ESI/LTQ-Orbitrap) *m*/*z*: [M]^3–^ Calcd for C_42_H_75_O_9_S_6_^3–^ 305.1251; Found 305.1237.

**Scheme 3:**
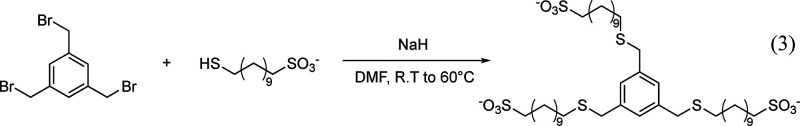
Synthesis of B3C11SO3

#### Synthesis of B3C6SO4 (the Same as the Synthesis of B3C11SO4)^22^

1,3,5-tris­(bromomethyl)-benzene and 6-mercapto-1-hexanol were used to synthesize compound B3C6OH. The product was a white powder (yield = 50.1%). 1H NMR (400 MHz, CDCl_3_) δ 7.08 (s, 3H, Ar–H), 3.62 (s, 6H, Ar–CH_2_), 3.53 (t, *J* = 6.6 Hz, 6H, CH_2_-O), 2.66 (s, 3H, OH), 2.35 (t, *J* = 7.4 Hz, 6H, S–CH_2_–C), 1.49 (dt, *J* = 11.2, 6.8 Hz,12H, C–CH_2_–C), 1.30 (dp, *J* = 12.5, 7.0, 6.5 Hz, 12H, C–CH_2_–C).13C NMR (101 MHz, CDCl_3_) δ139.00, 127.94, 62.51, 36.05, 32.50, 31.30, 29.16, 28.63, 25.35. HRMS (nanochip-ESI/LTQ-Orbitrap) *m*/*z*: [M + Na]+ Calcd for C27H48NaO3S3+ 539.2658; Found 539.2665.

From compound B3C6OH following the general method gave compound B3C6SO4 as a white powder, (yield= 67.5%).1H NMR (400 MHz, D_2_O) δ 7.19 (s, 3H, Ar–H), 4.07 (t, *J* = 6.6 Hz, 6H, CH_2_-O), 3.71 (s, 6H, Ar–CH_2_), 2.45 (t, *J* = 7.3 Hz, 6H, S–CH_2_–C), 1.69–1.38 (m, 24H, C–CH_2_–C).13C NMR (101 MHz, D_2_O) δ 139.19, 128.13, 69.35, 35.38, 30.91, 28.80, 28.64, 28.11, 24.82. HRMS (nanochip-ESI/LTQ-Orbitrap) *m*/*z*: [M]-2 Calcd for C27H45NaO12S6–2 388.0572; Found 388.0555.

#### Synthesis of B3C6SO3

Dissolve sodium 6-mercaptohexane-1-sulfonate (10 mmol) and Cs_2_CO_3_ (12 mmol) in 15 mL of CH_3_CN and stir at room temperature for 1 h. Add 1,3,5-Tris­(bromomethyl)­benzene (3 mmol) to the solution and stir at 55 °C for 18 h. Wash the product with a mixture of CH_3_CN and ethanol, then rinse with CH_3_CN 40%. Finally, the product was dried by centrifugation, then the surfactant was collected, and the solvent was removed by a vacuum drying (yield = 88%).^1^H NMR (400 MHz, D_2_O) δ 7.24 (s, 3H, Ar–H), 3.77 (s, 6H, Ar–CH_2_), 2.9–2.85 (t, 6H, CH_2_–SO_3_-), 2.45 (t, *J* = 7.7 Hz, 6H, S–CH_2_–C), 1.91 – 1.46 (m, 24H, CH_2_–CH_2_–CH_2_). HR-MS (nanochip-ESI/LTQ-Orbitrap) *m*/*z*: [M]^3–^ Calcd for C_27_H_45_O_9_S_6_^3–^ 235.0468; Found 235.0465.

#### Synthesis of C4C11SO4 (1D) and C4C11SO3 (2D)^23^

The syntheses of C4C11SO4 and C4C11SO4 are presented in Scheme 4. Dibenzo-18-crown-6 was functionalized with −CH_2_Br with a combination of paraformaldehyde and hydrogen bromide in CH_3_COOH, as reported to provide compound **1** in 80% yield. Subsequently, base (Cesium carbonate, Cs_2_CO_3_) assisted addition of 11-Mercaptoundecanol to produce compound **2** in high yield. This intermediate was further treated with sulfur trioxide-pyridine complex (SO_3_.Pyr) to provide sulfated derivative **1D** (yield = 48%). Similarly, compound **2D** was assessed by direct addition of 11-mercaptoundecane-1-sulfonate (yield = 20%). Compound 1D: ^1^H NMR (400 MHz, D_2_O) δ 7.00–6.8 (m, 7H), 3.33 (s, 3H), 2.88 (dt, *J* = 22.8, 10.8 Hz, 5H), 2.60 (s, 3H), 1.73 (s, 5H), 1.31 (s, 58H). HRMS (ESI/QTOF) *m*/*z*: [M]^−4^ Calcd for C_68_H_116_O_18_S_8_^–4^ 369.1487; Found 369.1482.

**Scheme 4:**
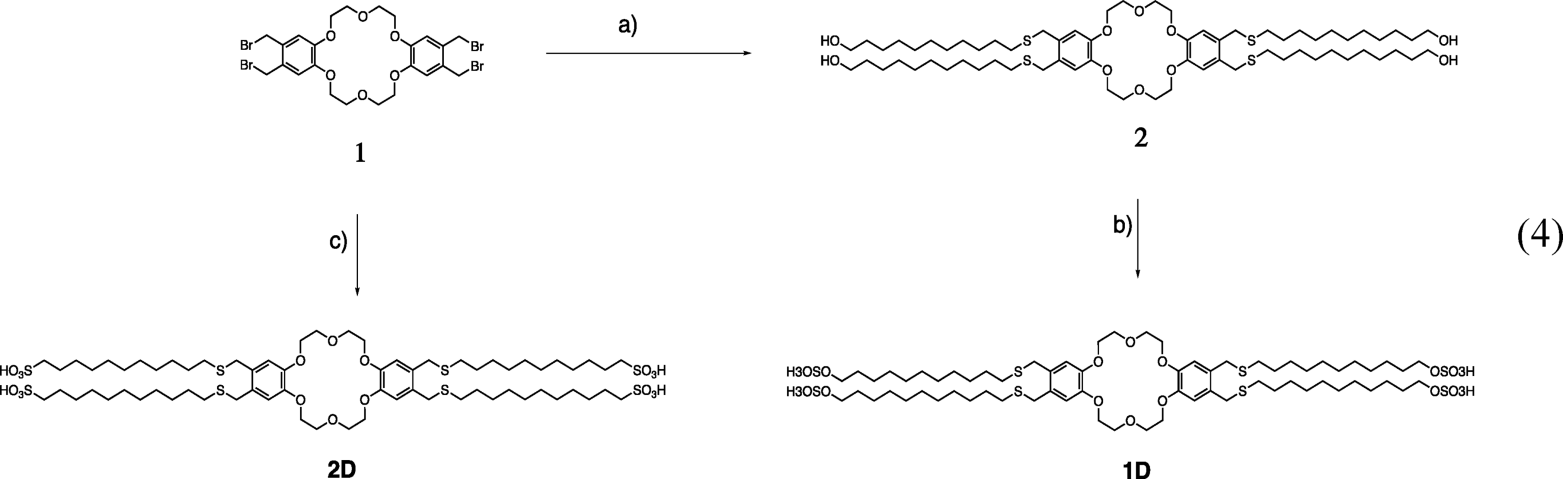
Synthesis of C4C11SO4 (**1D**) and C4C11SO3 (**2D**)­^a^

a Reaction conditions: (a) 11-Mercaptoundecanol, CS_2_CO_3_, DMF, 70 °C. (b) SO_3_·Py, DMF, 70 °C. (c) 11-mercaptoundecanesulfonate, Et_3_N, DMF, 60 °C.

##### Compound 2D

^1^H NMR (400 MHz, CDCl_3_) δ 9.38 (s, 6H), 8.66 (s, 8H), 8.32 (s, 6H), 7.48 (s, 3H), 4.15 (s, 28H), 3.09 (d, *J* = 49.5 Hz, 6H), 1.62 (d, *J* = 56.1 Hz, 8H), 1.32 (d, *J* = 6.9 Hz, 60H). ESI-MS: *m*/*z* (M-3H)^−3^ calcd for C_68_H_120_O_22_S_8_: 513.85; found: 513.85.

C = crown ether, 4 = four linkers, C11 = an 11-methylenes long linker, SO4 = sulfate, SO3 = sulfonate.

#### Synthesis of C4C3SO3

To a solution of C_24_H_28_O_6_Br_4_- compound 1 (500 mg, 0.713 mmol) in anhydrous DMF (20 mL) was added SH­(CH_2_)_3_SO_3_Na (1015.3 mg, 5.704 mmol) and NaH (274 mg, 11.4 mmol). The reaction mixture was stirred at 60 °C for 16 h under an inert atmosphere (argon or nitrogen). The mixture was extracted with hot ethanol to remove NaBr. The mixture was then purified by reversed-phase flash chromatography (C18, Methanol/water gradient) to afford the product (yield = 51%). HRMS (ESI/QTOF) *m*/*z*: [M]^−3^ Calcd for C_36_H_52_NaO_18_S_8_^–3^ 350.3611; Found 350.3603. ^1^H NMR (400 MHz, D_2_O) δ 6.83 (s, 4H, Ar–H), 4.06 (s, 8H, Ar–CH_2_), 3.61–3.88 (m, 16H, O–CH_2_–CH_2_–O), 2.6–2.63 (t, 8H, CH_2_–SO_3_), 2.06–2.1 (t, 8H, S–CH_2_), 1.8–1.99 (m, 8H, CH_2_–CH_2_–CH_2_).

#### HSV-2 Inhibition Assay

The inhibitory effect of compounds on HSV-2 was evaluated by plaque reduction assay. Vero cells were plated 24 h before the experiment in 24-well plates with a seeding density of 10^5^ cells/well. Serial dilutions of compounds in DMEM medium (2% FBS, 1% P/S) were prepared, followed by the addition of aliquots of HSV-2 such that the final titer of the virus was 200 pfu/mL (with MOI = 0.0005). Compounds/virus mixtures were incubated at 37 °C for 1 h and added to the cells (250 μL/well). Followed by incubation at 37 °C for 1 h, the viral inoculum was removed. The cells were overlaid with DMEM medium containing 1.2% methylcellulose, 2% FBS, and 1% P/S. After 48 h of incubation at 37 °C, 5% CO_2_, the cells were fixed and stained with 0.1% crystal violet in 20% ethanol. Finally, the plaques were counted. Each group of experiments in the inhibition assay was performed in triplicate. The concentration producing a 50% reduction in plaque formation was determined using Prism 10 software (GraphPad Software, USA) by comparing compound-treated and untreated cells. Infectivity was calculated as the number of plaques in compound-treated cells/number of plaques in untreated cells × 100%.

#### HSV-1 Inhibition Assay

The inhibitory effect of compounds on HSV-1 was evaluated by plaque reduction assay. Vero cells were plated 24 h before the experiment in 24-well plates at a density of 10^5^ cells/well. Serial dilutions of compounds in DMEM medium (2% FBS, 1% P/S) were prepared, followed by the addition of aliquots of HSV-1 such that the final titer of the virus is 400 pfu/ml (with MOI = 0.0005). Compounds/virus mixtures were incubated at 37 °C for 1 h and added to the cells (250 μL/well). Followed by incubation at 37 °C for 1 h, the viral inoculum was removed. The cells were overlaid with DMEM medium containing 1.2% methylcellulose, 2% FBS, and 1% P/S. After 3 days of incubation at 37 °C, 5% CO_2_, the cells were fixed and stained with 0.1% crystal violet in 20% ethanol, and plaques were counted. Each group of experiments in the inhibition assay was performed in triplicate. The concentration producing a 50% reduction in plaque formation was determined using the Prism 10 software (GraphPad Software, USA) by comparing compound-treated and untreated cells. Infectivity was calculated as the number of plaques in compound-treated cells/number of plaques in untreated cells × 100%.

#### Influenza Inhibition Assay

MDCK cells were preseeded in 96-well plates and allowed to adhere for 24 h. Dilutions of the compound were prepared in DMEM supplemented with 1% P/S and mixed with the Influenza A/Netherlands/602/2009 (H1N1), H1N1 A/Lausanne/2022 clinical, H3N2 A/Wyoming/2003/3, B/Washington/02/2019 MOI = 0.01 at 37 °C for 1 h. The cell mixture was then added to the preplated cells. After 1 h of virus adsorption at 37 °C, the inoculum was removed, and fresh DMEM or MEM medium with 1% P/S was added. Following 24 h of incubation at 37 °C, an immunocytochemical (ICC) assay was performed to analyze the infection of the influenza virus. The cells were fixed and permeabilized with methanol for 1 min, incubated with Flu A monoclonal antibody with influenza A and flu B monoclonal antibody for influenza B (1:2000 dilution-A, 1:50 dilution-B) for 1 h at 37 °C, washed with a washing buffer (PBS + Tween 0.05%) for three times, and incubated with gout antimouse IgG- Alexa fluor 488 (1:1000 dilution) for 1 h. The cells were washed with PBS three times, and then DAPI was added for 5 min. The cells were then washed with PBS/Tween 0.05% three times. Fluorescent infected cells were automatically counted by using a plate reader, and the percentages of infection were calculated by comparing the number of infected cells in treated and untreated conditions. The infectivity percentage was presented as mean ± SD, *n* = 3. The effective concentrations IC50 were calculated by nonlinear regression analysis [log­(inhibitor) versus response – variable slope (four parameters)] in GraphPad Prism 10 (GraphPad Software, USA).

#### HSV-2 Virucidal Assay

Virucidal effects were evaluated by performing viral titration on dilutions of compounds/virus mixtures. Vero cells were plated 24 h before the experiment in 24-well plates at a density of 10^5^ cells/well. The 90–99% viral inhibition concentrations of the compounds were applied for virucidal titration to examine the (ir)­reversibility of the inhibition, and HSV-2 viruses (MOI = 1.5) were incubated at 37 °C for 1 h. Serial dilutions of compounds/virus mixtures were prepared and transferred to cells (250 μL/well). Followed by incubation at 37 °C for 1 h, the viral inoculum was removed. The cells were overlaid with DMEM medium containing 1.2% methylcellulose, 2% FBS, and 1% P/S. After 48 h of incubation at 37 °C, 5% CO_2_, the cells were fixed and stained with 0.1% crystal violet in 20% ethanol, and plaques were counted. Viral titers were calculated at dilutions at which the compound was not effective. Each group of experiments in the virucidal assay was performed in triplicate.

#### HSV-1 Virucidal Assay

Virucidal effects were evaluated by performing viral titration on dilutions of compounds/virus mixtures. Vero cells were plated 24 h before the experiment in 24-well plates at a density of 10^5^ cells/well. The 90–99% viral inhibition concentrations of the compounds were applied for virucidal titration to examine the (ir)­reversibility of the inhibition, and HSV-1 viruses (MOI = 1.5) were incubated at 37 °C for 1 h. Serial dilutions of compounds/virus mixtures were prepared and transferred to the cells (250 μL/well). Followed by incubation at 37 °C for 1 h, the viral inoculum was removed. The cells were overlaid with DMEM medium containing 1.2% methylcellulose, 2% FBS, and 1% P/S. After 3 days of incubation at 37 °C, 5% CO_2_, the cells were fixed and stained with 0.1% crystal violet in 20% ethanol, and plaques were counted. Viral titers were calculated at dilutions at which the compounds were not effective. Each group of experiments in the virucidal assay was performed in triplicate.

#### Influenza Virucidal Assay

The 90–99% viral inhibition concentrations of the compounds were applied for virucidal titration to examine the (ir)­reversibility of the inhibition. Each influenza virus (MOI = 0.5) was incubated at 37 °C for 1 h. Subsequently, the resulting complex of viruses and materials, as well as the untreated control, underwent serial dilution. These diluted samples were then transferred onto MDCK cells and left for 1 h. After that, the mixture was removed, and a fresh DMEM medium with 1% P/S was introduced. The next day, the viral titers were evaluated using the ICC assay, which was previously described.

#### Cytotoxicity Assay on Vero Cells

Cytotoxicity assay was performed on the Vero cell line with the MTS assay. The cells were plated for 24 h in DMEM medium containing 10% FBS and 1% penicillin/streptomycin with a seeding density of 2 × 10^4^ per well in a 96-well plate. Tested compounds were serially diluted in the identical medium (2% FBS, 1% P/S), added to the cells, and incubated for 48 h (the same experimental condition for IC_50_ of HSV-2). Then, the cells were washed with PBS twice, followed by the addition of 20 μL MTS reagents together with 80 μL DMEM medium. They were then incubated at 37 °C for 4 h. The cell viability was checked by absorbance at 490 nm with a plate reader.

#### Cytotoxicity Assay for MDCK

MTT assay was used to evaluate the cytotoxicity on MDCK cells. 10^4^ cells per well were seeded in a 96-well plate 1 day before the assay. A dose range of each drug (from 4.11 μM to 1000 μM) was added to the cells in serum-free medium for MDCK cells. The antivirals were incubated on the cells for 24 h at 37 °C. After incubation, the cells were washed, and MTT reagent (Promega) was added to the cells for 4 h at 37 °C according to the manufacturer’s instructions. The cells were then washed, and 50 μL of DMSO was added to free the reagent. Subsequently, the absorbance was read at 579 nm. Percentages of viability were calculated by comparing the absorbance in treated wells and untreated conditions.

#### Growth of Viruses in the Presence of Compounds (Posttreatment)

The inhibitory effect of compounds on HSV-2 was evaluated by a plaque reduction assay. Vero cells were plated 24 h before the experiment in 24-well plates with a seeding density of 10^5^ cells/well. Serial dilutions of compounds in DMEM medium (2% FBS, 1% P/S) were prepared, followed by the addition of aliquots of HSV-2 incubated with cells at 37 °C for 1 h (with MOI = 0.0005). Followed by incubation at 37 °C for 1 h, the viral inoculum was removed. After that, different concentrations of MEIs were added and incubated at 37 °C for 1 h. Subsequently, all of the supernatants were removed. The cells were overlaid with DMEM medium containing 1.2% methylcellulose, 2% FBS, and 1% P/S. After 48 h of incubation at 37 °C, 5% CO_2_, the cells were fixed and stained with 0.1% crystal violet in 20% ethanol. Finally, the plaques were counted. Each group of experiments in the inhibition assay was performed in triplicate. The concentration producing a 50% reduction in plaque formation was determined using Prism 10 software (GraphPad Software, USA) by comparing compound-treated and untreated cells. Infectivity was calculated as the number of plaques in compound-treated cells/number of plaques in untreated cells × 100%.

#### CMC Assay

The CMC of antiviral compounds was measured by the conductivity method. Antiviral compounds were diluted into water at different concentrations, and the conductivity (μS/cm) was measured at each concentration with a conductivity meter (Mettler Toledo, Switzerland). The CMC was identified as the point on the conductivity-concentration plot where the slope changed.

#### Partition Coefficient

UV–vis spectroscopy was employed first to determine the correct dilution of all compounds. The seven compounds in this study were B3C11SO4, B3C11SO3, B3C6SO3, B3C6SO4, C4C11SO4, C4C11SO3, and C4C3SO3. The protocol is the same for all the compounds: in the beginning, all compounds were dissolved in water at a concentration of 1 mg/mL. The solution was put into a quartz cuvette and then into the UV–vis spectrometer, where the absorbance from wavelengths 600 to 190 nm was investigated. All of the samples presented a peak in absorbance around 200 nm. The samples were diluted further until the absorbance peak reached values near 0.90.

A biphasic solution composed of 1.5 mL of a water solution of antiviral +1.5 mL of octanol was thoroughly mixed for 60 min. After the mixing, the two phases were separated by centrifugation for 1 min using a benchtop centrifuge at 5000 rpm. The water phase saturated in octanol and the octanol phase saturated with water were extracted and characterized by UV–vis spectrometry.

The partition coefficient (log *P*) was then calculated using formula [Disp-formula eq1]:

$$\log P=\log\left(\frac{\mathrm{Absorbance}\;\mathrm{of}\;\mathrm{octanol}\;\mathrm{phase}}{\mathrm{Absorbance}\;\mathrm{of}\;\mathrm{water}\;\mathrm{phase}}\right)$$ 1

#### Isothermal Titration Calorimetry assay

Isothermal titration calorimetry (ITC) experiments were conducted using ITC200 instruments (GE Healthcare, USA) equipped with a 200 μL sample cell and a 40 μL syringe. Thirteen injections were applied. The first data point was excluded. BSA protein and viral protein concentrations in the cell were approximately 50 μM and 1 mg/mL, respectively, and ligand concentrations in the syringe were roughly 2 mM. They were centrifuged at 2000*g* for 2 min to remove bubbles. The titration was performed at 25 or 37 °C, with data analyzed using MicroCal PEAQ ITC software. Baseline correction was applied, and Nitpic/Sedphat was used for automated integration to ensure precise binding parameter determination.

## Results

The names, abbreviations, and chemical structures of all MEI compounds are tabulated in Table 1. The chemical synthesis is shown in the Materials and Methods section. The results of the virucidal assay are shown in Table 2, and virucidal assay curves are presented in Figures SI7–SI12. The values of IC_50_ are presented in Table 3, and inhibition curves are shown in Figures SI1–SI6.

**Table 1:**
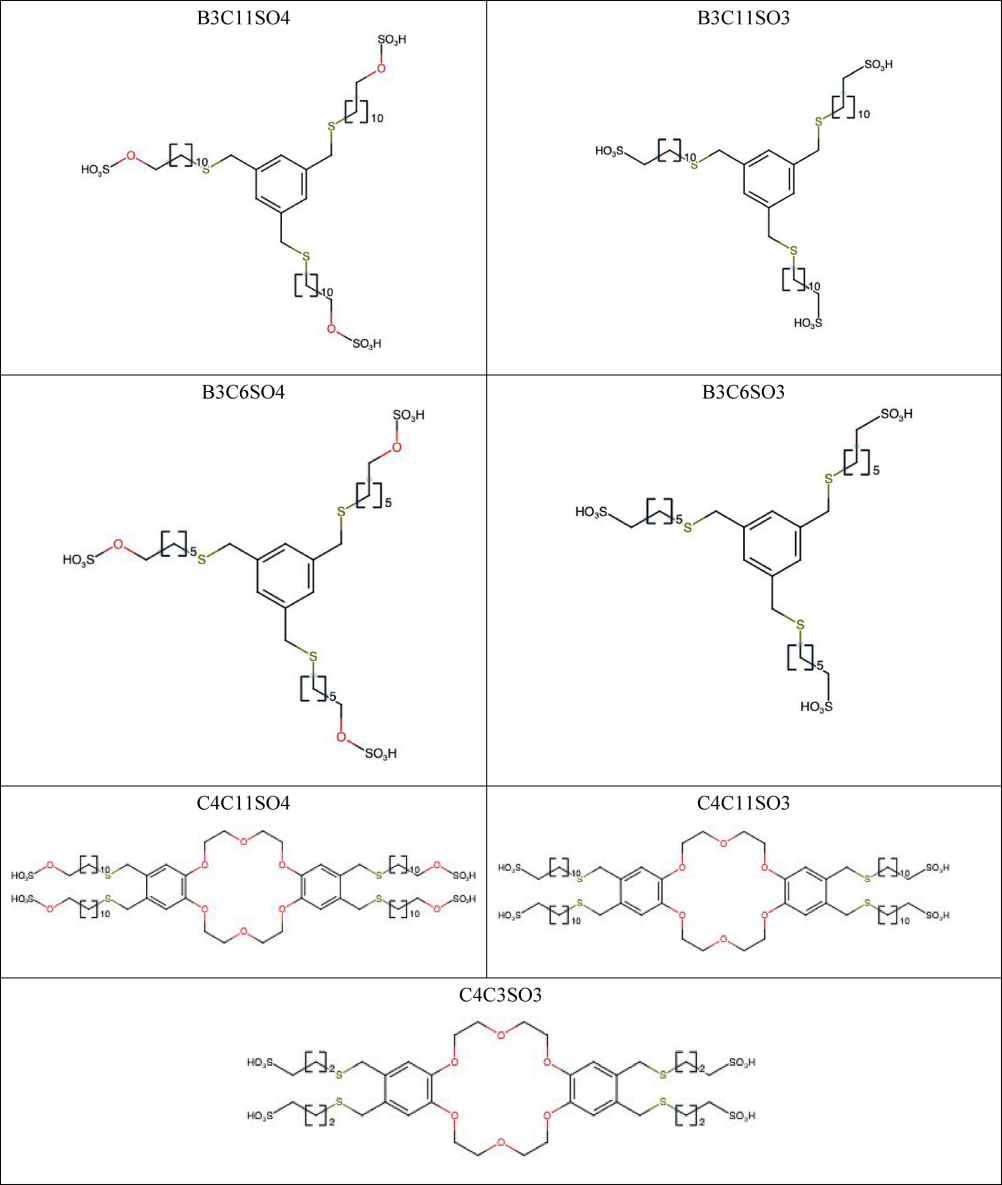
Name Abbreviation and Structure of Antiviral Compounds

**Table 2:** Virucidal Activity of B3C11SO4 and B3C11SO3 on HSV and Influenza Inhibition^a^

	V-1	HSV-2	H1N1N09	H1N1 clinical	H3N2	FluB
B3C11SO4	V	V	V	V	V	V
B3C11SO3	S	S	S	S	S	S
B3C6SO4	V	V	V	NA	NA	NA
B3C6SO3	S	S	NA	NA	NA	NA
C4C11SO4	V	V	V	V	V	NA
C4C11SO3	V	V	V	V	V	V
C4C3SO3	S	S	NA	NT	NA	NA

a V: Virucidal, S: Virustatic, NA: No virucidal Activity, NT: Not Tested.

**Table 3:** IC_50_(μM) Values of Antiviral Compounds Obtained from Their Dose–Response Curves on HSV and Influenza Inhibition Assay^a^

compounds	HSV-1	HSV-2	H1N1N09	H1N1 clinical	H3N2	Flu B
B3C11SO4	43 ± 1.14	43 ± 8.3	0.9 ± 0.05	0.7 ± 0.04	0.6 ± 0.12	0.4 ± 0.04
B3C11SO3	138.9 ± 4.6	88.5 ± 7.6	35.17 ± 4.7	4.2 ± 0.36	49.5 ± 8.7	324 ± 103
B3C6SO4	208.3 ± 31.37	52.9 ± 1.9	346.9 ± 1.94	NA	NA	NA
B3C6SO3	502.3 ± 2.15	487.3 ± 2.19	NA	NA	NA	NA
C4C11SO4	22.5 ± 4.2	9.8 ± 1.5	80.9 ± 13.69	>202.4	40.9 ± 1.19	47.9 ± 9.38
C4C11SO3	118.4 ± 10.4	18.5 ± 5.2	15.8 ± 3.1	10.4 ± 3.34	6.4 ± 1.03	7.7 ± 3.75
C4C3SO3	551.9 ± 2.0	173.36 ± 1.1	NA	NT	NA	NA

a NA: *N*o inhibition *A*ctivity, NT: *N*ot *T*ested.

Virucidal tests confirmed that B3C11SO4 exhibits virucidal activity, whereas B3C11SO3 functions as a virustatic agent against HSV-2, as reported in our recent study.^22^ To evaluate the broad-spectrum antiviral properties, we investigated whether the observed distinction in the virucidal activity against HSV-2 was the same in other viruses. We tested these antivirals with HSV-1 and four other types of influenza viruses (H1N1N09- H1N1 Netherlands 2009, H1N1 Clinical, H3N2, and FluB). Across all these viruses, for the benzene core, it is consistent that the sulfate MEI is virucidal and that the sulfonate MEI is virustatic. This consistency is maintained for sulfate and sulfonate derivatives with a shorter linker, B3C6SO4 and B3C6SO3. To assess the role of the core structure in relation to virucidal activity, we examined C4C11SO4 and C4C11SO3 MEIs.^23^ Unlike MEIs based on benzene cores, both C4C11SO4 and C4C11SO3- with identical linkers but different functional groups- demonstrate virucidal activity. Interestingly, C4C3SO3, an MEI with a crown ether core and a shorter linker (C3-sulfonate), is virustatic.

We studied the inhibition mechanisms of virucidal and virustatic antivirals by comparing their pretreatment and posttreatment conditions. The results show that for virustatic compounds, the IC_50_ in posttreatment was consistently lower than in pretreatment. As for the virucidal compounds, no clear relationship between the two values could be found (Table 4). The inhibition curves are shown in Figures SI15 and SI16.

**Table 4:** IC_50_ (μM) Values of Viral Inhibition under Pretreatment and Posttreatment Conditions, Respectively^a^

viruses	MEIs	pretreatment IC_50_	posttreatment IC_50_
**HSV-2**	B3C11SO4	43.0 ± 8.3	18.0 ± 1.13
	B3C11SO3	88.5 ± 7.6	10.3 ± 1.17
	B3C6SO4	52.9 ± 1.9	56.9 ± 1.48
	B3C6SO3	487.3 ± 2.19	180.7 ± 2.06
	C4C11SO4	18.5 ± 5.2	4.8 ± 0.7
	C4C11SO3	9.8 ± 1.5	12.3 ± 0.76
	C4C3SO3	173.36 ± 1.1	69.14 ± 1.01
**H3N2**	B3C11SO4	0.6 ± 0.12	8.6 + 1.16
	B3C11SO3	49.5 ± 8.7	24.8 ± 1.2
	B3C6SO4	346.9 ± 1.94	NT
	B3C6SO3	NA	NT
	C4C11SO4	6.4 ± 1.03	18.3 ± 0.75
	C4C11SO3	40.9 ± 1.19	52.6 ± 0.5
	C4C3SO3	NA	NT

a NA: *N*o antiviral *A*ctivity, NT: *N*ot *T*ested.

To evaluate the anti-inflammatory potential of MEIs, we investigated their ability to reduce the level of NO production in macrophages. RAW 264.7 cells were pretreated with the different concentrations of MEIs for 2 h, followed by stimulation with lipopolysaccharide (LPS) (1 μg/mL) for 24 h to induce inflammation. As expected, LPS treatment significantly increased the level of NO synthesis. However, cotreatment with MEIs led to a reduction in NO levels, confirming their anti-inflammatory effect. Notably, virucidal MEIs significantly inhibited NO production in LPS-stimulated macrophages, with B3C11SO4 showing an IC_50_ of 152.8 ± 1.27 μM, C4C11SO3 at 173.8 ± 1.52 μM, and C4C11SO4 66.11 ± 1.23 μM. In contrast, virustatic compounds exhibited no inhibitory effect.

Next, we investigated the binding of MEI to proteins. Table SI4 shows no significant difference in *K*_d_ values between B3C11SO4 and B3C11SO3 with HA1 from the influenza virus. The MEIs with shorter linkers (B3C6SO4 and B3C6SO3) were found to exhibit weaker binding, and the sulfate MEI demonstrated stronger binding than the sulfonate one (see Table SI4). As for derivatives of crown ether, C4C11SO4 has stronger binding to HA1 than C4C11SO3. *K*_d_ measurements between glycoprotein D (gD) of HSV and MEIs do not show the same trend. Sulfate MEI B3C11SO4 binds more weakly than sulfonate B3C11SO3. The same conclusion was also found for both pairs B3C6SO4 and B3C6SO3, and the pair C4C11SO4 and C4C11SO3 (Table SI4). Our results suggest that there is no consistent trend in binding between virucidal and virustatic compounds with viral proteins. We, therefore, proposed BSA as a model protein to evaluate how virucidal and virustatic compounds interact with protein surfaces. The BSA surface is composed of both hydrophobic and charged patches, which allows us to examine the contribution of each type of interaction.^24^ We considered the roles of multivalency, functional groups, linker length, and thermodynamic energies in the interactions between virucidal compounds and BSA. B3C11SO4 and B3C11SO3 exhibit approximately 1 binding site with BSA, with the former binding more strongly (Table 5). In our study, sulfate-based virucidal compounds showed stronger BSA binding than sulfonate-based virustatic compounds. In addition, the compounds with shorter linker lengths (B3C6SO3 and B3C6SO4) corresponded to weaker interactions with BSA. Overall, benzene-base MEIs exhibited lower *K*_d_ values than crown ether-derived MEIs.

**Table 5:** Dissociation Constant *K*_d_ of Antivirals and BSA in H_2_O at 25 °C

compounds	*K*_d_ (μM)	Δ*H* (kcal/mol)	Δ*G* (kcal/mol)	–ΔTS (kcal/mol)	*N*
B3C11SO4	2.23	–5.4	–7.71	–2.31	1.1
B3C11SO3	5.82	–9.08	–7.15	1.93	1.1
B3C6SO4	12.2	–8.93	–7.99	0.938	1.3
B3C6SO3	no binding				
C4C11SO4	35.7	–2.73	–6.07	–3.34	2.5
C4C11SO3	10.3	–0.934	–6.81	–5.88	2.63
C4C3SO3	63	–7.34	–5.73	1.61	0.5

In an attempt to correlate the molecules’ inhibition mechanism with their properties, we measured a series of key physical chemistry properties that are linked with hydrophobic effects. We determined the CMC of all compounds in order to determine whether molecular aggregates have any contribution to the virucidal properties. At concentrations larger than the CMC, molecules are presumed to have, at least in part, micellar aggregates in solution. The results are tabulated in Table 6. Together with IC_99_ (concentrations of antivirals used in experiments intended to inhibit infection by at least 90 and 99%), and CC_50_ (the concentration of test compounds required to reduce cell viability by 50%) (see Figures SI13, SI14, and Table SI1), CMCs are used to understand the relationship between compound properties and antiviral efficacy (Table 6). All compounds have the CMC values higher than IC_50_ (Tables 3 and 6). Most compounds have IC_99_ values smaller than their CMC values, except for B3C11SO3 and C4C11SO3 when tested with HSV-2. Further measurements of virucidal properties at three different concentrations (lower, equal to, and higher than the CMC, all below the CC_50_) for C4C11SO3 and B3C11SO3 showed that C4C11SO3 is virucidal, while B3C11SO3 is virustatic, regardless of the tested concentrations (Table SI2).

**Table 6:** CMC and IC_99_ of Antiviral Compounds in HSV-2 Inhibition

compounds	CMC (μM)	IC_99_ (μM)
B3C11SO4	556 ± 35.1	103.8 ± 8.3
B3C11S03	497 ± 20.9	1092 ± 7.6
B3C6SO4	1390 ± 45.6	1328 ± 1.9
B3C6SO3	882.2 ± 18.9	
C4C11SO4	1003 ± 24.6	67.47 ± 5.2
C4C11SO3	237 ± 17.1	408 ± 1.5
C4C3SO3	462.69 ± 57	446 ± 1.1

We further evaluated the hydrophobicity of the antiviral compounds by measuring their partition coefficients (log*P*) in the biphasic liquid–liquid system of octanol and water. The partition coefficient was measured via a UV–vis assay, and the result was presented in Table 7. It shows that in general, virucidal compounds are more hydrophobic than virustatic ones.

**Table 7:** Partition Coefficients of MEIs by Means of Log*P* Measurement and Theoretical Calculation of Hydrophilic and Lipophilic Balance (HLB) Scores

compounds	Log*P*	HLB score
B3C11SO4	–2.2 ± 0.07	103.15
B3C11S03	–2.43 ± 0.09	20.05
B3C6SO4	–1.79 ± 0.08	110.275
B3C6SO3	–2.39 ± 0.1	27.175
C4C11SO4	–1.76 ± 0.06	137.3
C4C11SO3	–2.25 ± 0.05	26.5
C4C3SO3	–2.45 ± 0.04	41.7

## Discussion

Recent studies in our laboratory have shown that stoichiometric sulfonate and sulfate compounds, as multivalent inhibitors, show tremendous potential for being potent antivirals.^23^ More importantly, the use of stoichiometric compounds facilitates direct comparison of the antiviral properties and accordingly helps dissect precisely the complex interplay between molecular features and virucidal efficacy. The key factors that have been investigated include the number of ligands, ligand length, core type, and core size.^22^ Our recent observation showed that two benzene core-derived MEIs (B3C11SO4 and B3C11SO3) demonstrated distinct antiviral mechanisms, i.e., against HSV-2, B3C11SO4 being virucidal and B3C11SO3 being virustatic, respectively. This distinction remains for HSV-1 and four other types of influenza in this study, suggesting that it is a general phenomenon. Previously, it was demonstrated that linker length strongly affects antiviral mechanism and efficacy.^22^ The general understanding is that for virucidal compounds with shorter linkers, they have weaker affinity to viral proteins, thus being less effective and could become virustatic. However, for all viruses tested, B3C6SO4 was found to be virucidal and B3C6SO3 virustatic, similar to the pair of MEIs with C11 linker. Therefore, our current study highlights how subtle molecular features, especially functional group chemistry for benzene cores, drive distinct antiviral mechanisms across different viral families.

We continued to investigate the influence of core types on the virucidal properties by keeping the functional groups (sulfate and sulfonate) and the length of the linker. Therefore, we synthesized the new pair: C4C11SO4 and C4C11SO3. Interestingly, unlike the pairs based on a benzene core, both of these MEIs are virucidal. This indicates that with crown ether cores and C11 linker length, the functional groups do not significantly affect the inhibition mechanism. To further assess the role of linker’s length associated with crown ether cores in virucidal activity, we showed that C4C3SO3 is virustatic. These results suggest that molecular hydrophobicity plays a direct and crucial role in determining the differences in the virucidal properties.

To elucidate what affects the antiviral distinction among these pairs of compounds, we measured the binding of MEIs with viral proteins related to viral entry, such as glycoprotein D (gD) of HSV and hemagglutinin (HA) of influenza. HSV glycoprotein gD plays an important role in viral entry by specifically binding to membrane receptors such as HVEM, nectin-1, and 3-O-sulfated heparan sulfate.^25^ Therefore, gD represents an ideal target for antiviral strategies. HA is the main surface glycoprotein of influenza viruses and belongs to the class I fusion protein family.^26^ HA is a mushroom-shaped homotrimer, initially expressed as a precursor (HA0) that is cleaved by host proteases into two disulfide-linked subunits: HA1 and HA2. HA1 forms the globular head and contains the receptor-binding site (RBS), which recognizes sialic acid residues on host membrane glycoproteins.^26^ HA2 makes up the stem region of HA and is highly conserved across subtypes due to the presence of the N-terminal fusion peptide.^26^ Due to their essential roles in viral entry, both the RBS of HA1 and the fusion peptide of HA2 are attractive targets for antivirals. In this study, similar binding patterns were not observed for HA1 or gD with these MEIs.

We employed a model protein (BSA) and studied its interaction with all of the antivirals, owing to the advantage that all of the MEIs exhibit broad-spectrum antiviral activity. It is worth pointing out that, as a globular, nonglycosylated protein with a three-dimensional structure similar to human serum albumin (HSA), it has approximately 80% sequence homology and 76% structural similarity.^27^ HSA is the most abundant plasma protein in the human body, constituting about 60% of total plasma proteins.^28^ It plays an important role in transporting a broad range of endogenous and exogenous substances, including hormones, fatty acids, and drugs.^29^ BSA serves as an ideal model system for in vitro studies of drug–protein interactions, hence it is well-suited for our purpose.^30–33^ Most importantly, BSA exposes on its surface patches of hydrophobic and charged nature; as such, it allows us to explore and dissect the contribution of each interaction component. We excluded the significance of charge interaction in our case, as none of the compounds bound to lysozyme at a measuring pH of 7.4, where lysozyme has a net positive charge. This result suggests that the interaction between the antivirals under investigation with BSA could be explained by a patchy interaction model.^34^ Increasing the temperature from 25 to 37 °C under the same buffer conditions (water and PBS) decreased *K*_d_ values for all compounds, suggesting that hydrophobic interactions play a key role in the binding. Indeed, our results demonstrated that virucidal compounds exhibit stronger hydrophobic interactions with BSA compared to virustatic compounds, driven by their sulfate functional groups and optimal linker designs (Table SI3). These findings offer important insights into how the structure and energy properties of antivirals influence their activity.

The CMC and hydrophobicity of the compounds are closely linked to their antiviral efficacy. Mehznaz et al. showed that gemini surfactants with longer hydrocarbon chains exhibited greater hydrophobicity and correspondingly lower CMC values, which translated to higher antiviral activity against H1N1, and the hydrophobicity of compounds enhances the compound’s ability to interact with viral components like surface glycoproteins or membranes.^35^ Such interactions likely disrupt viral attachment and entry into host cells. In contrast, compounds with short chains and rigid spacers showed higher CMCs and reduced antiviral effects.^35^ In another study on linear alkylbenzenesulfonates, it was shown that the CMC reduces as the length of the linker increases.^36^ This property is similar to what is observed with the compounds used in this study. Therefore, the lower CMC generally indicates a greater molecular hydrophobicity. The trend is not always true. CMCs of alkyl sulfates were shown to be lower than their sulfonate counterparts only up to the chain length of 12 carbons.^37^ In our study, we observed no direct link between CMC and the antiviral exhibition mechanism. Furthermore, our CMC data exclude the role of molecular aggregation (i.e., micelles) in the concentration range of this study. The antiviral activity of MEIs is therefore primarily influenced by molecular features rather than aggregation status.

We further evaluated the hydrophobicity of the antiviral compounds by measuring their partition coefficients, which are related to the compounds' molecular structure. Virucidal compounds are found to be more lipophilic, in contrast to virustatic ones, which are more hydrophilic. This difference in hydrophilicity and lipophilicity highlights distinct mechanisms of action between virucidal and virustatic compounds. Based on these conclusions, we first associate the low hydrophobicity of virustatic compounds with their higher efficacy in the posttreatment, as these antivirals tend to perform better compared to pretreatment. For example, with HSV-2, B3C11SO3 shows a remarkable reduction in IC_50_, decreasing by approximately 9-fold, while B3C6SO3 exhibits a substantial decrease in IC_50_, dropping from 487 to 180 μM, as shown in Table 4. Similar trends are observed with H3N2 influenza, whereas virucidal antivirals do not clearly follow any pattern between pretreatment and posttreatment. virustatic compounds are generally less hydrophobic, which facilitates their solubility and intracellular diffusion since the cytoplasm is primarily a hydrophilic environment.^38^ This physicochemical property enables them to effectively reach and interact with viral enzymes inside host cells during posttreatment. In contrast, the hydrophobic interaction plays a dominant role in the binding of antivirals to viral surface proteins involved in attachment and entry. These proteins present exposed hydrophobic domains, making them susceptible to disruption by highly hydrophobic virucidal compounds. Although viral enzymes are also proteins, their active sites are highly specific and structurally constrained. Therefore, selective inhibition of enzymatic function requires not only hydrophobic interactions but also a combination of hydrogen binding, ionic interactions, and van der Waals forces. Compared to entry-related proteins, the contribution of hydrophobic interaction to antiviral enzyme binding is less dominant and must be complemented by additional interaction types to ensure selectivity and potency.^39^

All virucidal antivirals inhibited the production of NO, which is produced by macrophages in response to cytokines, microbial components, is synthesized from the amino acid l-arginine through the action of inducible nitric oxide synthase (iNOS or NOS2).^40^ Moreover, these compounds did not affect TNF-α or IL-6, it is likely to act directly on iNOS or its specific regulatory factors, rather than blocking the entire inflammation pathway such as NF-κB, which controls the expression of TNF-α, IL-6.^41^ In this study, we can consider the relationship between the hydrophobicity of virucidal compounds and the production of NO; whereas the mentioned virustatic compounds which are more hydrophilic, are not able to reduce the production of NO. The role of iNOS in infectious diseases, for example, influenza viruses, is detrimental to the host.^42^ iNOS forms a zinc-bridged homodimeric quaternary structure, enabling the enzyme to catalyze the conversion of l-arginine to L-citrulline, accompanied by the simultaneous production of NO.^43^ l-arginine is the substrate for iNOS via hydrogen bonds, electrostatic interactions, hydrophobic interactions, etc.^43^ It is possible that virucidal compounds interact with l-arginine through hydrophobic interactions, which could potentially reduce the level of interaction between iNOS and l-arginine.

Overall, due to their higher hydrophobicity, virucidal MEIs tend to exhibit stronger hydrophobic interactions with BSA, which will then influence their distribution, stability, and overall mechanism of action. Therefore, both the hydrophobicity of virucidal antivirals and their ability to engage in hydrophobic interaction with proteins appear to play a significant role in determining their virucidal properties.

## Conclusion

In this article, we present an in-depth study of antiviral inhibition mechanisms of pairs of MEIs whose difference is only in the terminal functional groups of the linkers. Sulfonate MEIs are virustatic, while sulfate ones are virucidal for benzene derivatives. This distinction disappears for crown ether-based MEIs, where both derivatives are virucidal. We further demonstrated that the length of linkers plays a key role in determining the virucidal mechanism. All of the sulfate compounds in this study were found to be virucidal.

Virucidal properties of MEIs were shown to correlate, consistent with the molecular hydrophobicity and hydrophobic interaction with proteins. Comparative partition coefficient analysis revealed that not only were virucidal sulfate compounds more hydrophobic than the virustatic sulfonate counterparts, but also all virucidal compounds were found to be more hydrophobic than virustatic ones. Virucidal compounds exhibit stronger hydrophobic interactions with BSA than virustatic compounds. As a result, virucidal compounds inhibit NO production, while more hydrophilic virustatic compounds do not exhibit this effect. In addition, the hydrophilicity of virustatic compounds likely contributes to their enhanced efficacy in the posttreatment conditions, as it facilitates intracellular diffusion and enables better access to viral enzymes within the host cells.

While our study highlights the importance of hydrophobic effects in enabling MEIs to act as broad-spectrum virucidals, the specific roles of functional groups such as SO_3_ versus SO_4_ in the inhibition mechanism are unresolved. The complex interplay between the cores, linkers, and functional groups makes it challenging to isolate the effect of an element. Nevertheless, the detailed observations presented in this work provide a foundation for future investigations aimed at elucidating how these functional groups influence virucidal activity.

## Supplementary Material



## Notes

All materials and methods, the results of ^1^H NMR, ^13^C NMR, dose response plots, virucidal assays, CC50, CMC, posttreatment assays, *K*_d_, and partition coefficient data, are found in the supporting materials (PDF)

## Abbreviations

MEIs: multivalent entry inhibitors
IC_50_: inhibition concentration
CMC: critical micellar concentration
NMR: nuclear magnetic resonance
ACs: antiviral compounds
CC_50_: cytotoxicity concentration
V: virucidal
S: virustatic
