# Supplementary material for: A Comparative Study of Virucidal and Virustatic Multivalent Entry Inhibitors
Source: J Phys Chem B. 2025 Oct 28;129(44):11453–64. doi: 10.1021/acs.jpcb.5c05864 (PMC12598866; doi:10.1021/acs.jpcb.5c05864)
Supplement: Supplementary file 1 [file jp5c05864_si_001.pdf]

Supporting Information

A Comparative Study of Virucidal and Virustatic Multivalent Entry Inhibitors

Hien Thi Tran[1], Sujeet Pawar[1], Yong Zhu[1], Quy Khac Ong[1*], Francesco Stellacci[1,2,3*]

[1] Institute of Materials, École Polytechnique Fédérale de Lausanne, Station 12, CH-1015 Lausanne, Switzerland.

[2] Institute of Bioengineering, École Polytechnique Fédérale de Lausanne (EPFL), Station 12, CH-1015 Lausanne, Switzerland.

[3] Global Health Institute, École Polytechnique Fédérale de Lausanne (EPFL), Station 12, CH-1015 Lausanne, Switzerland.

KEYWORDS: Antivirals, virucidal, virustatic, broad-spectrum antivirals, multivalent entry inhibitors.

* Corresponding authors: quy.ong@epfl.ch

francesco.stelacci@epfl.ch

Table of contents

1. Supplementary figures and tables 7

1.1. Viral inhibition assays performed with MEIs 7

1.2. The virucidal assays 13

1.3. The cytotoxicity assays performed with MEIs 19

1.4. The inhibition assays of MEIs in the growth of viruses 22

1.5. The relationship between CMC and virucidal properties 24

1.6. Dissociation constant of interactions between MEIs and BSA protein 25

1.7. Dissociation constant of interactions between MEIs and viral proteins 26

2. The results of synthesis 27

References 42

Figures

Figure S1. (a)Dose–response assay curves of HSV-1 in the presence of B3C6SO4 and B3C6SO3; (b) dose–response assay curves of HSV-1 in the presence of C4C11SO4 and C4C11SO3, c) dose–response assay curves of HSV-1 in the presence of B3C11SO4 and B3C11SO3; d) dose–response assay curves of HSV-1 in the presence of C4C3SO3 on Vero cells. Viral infection (%) was plotted against compound concentration (µg/ml) to assess antiviral activity. 7

Figure S2. a) Dose–response assay curves of HSV-2 in the presence of B3C11SO4 and B3C11SO3; b) dose–response assay curves of HSV-2 in the presence of B3C6SO4 and B3C6SO4; c) dose–response assay curves of HSV-2 in the presence of C4C3SO3; d) dose–response assay curves of HSV-2 in the presence of C4C11SO4 and C4C11SO3 on Vero cells. Viral infection (%) was plotted against compound concentration (µg/ml) to assess antiviral activity. 8

Figure S3. a) Dose–response assay curves of H1N1 Clinical of C4C11SO4 and C4C11SO3; b) dose–response assay curves of H1N1 Clinical of B3C11SO4 and B3C11SO3 on MDCK cells. Viral infection (%) was plotted against compound concentration (µg/ml) to assess antiviral activity......... 9

Figure S4. a) Dose–response assay curves of H1N1 NO9 of B3C11SO3 and B3C11SO4; b) dose–response assay curves of H1N1 NO9 of C4C11SO3 and C4C11SO4 on MDCK cells. Viral infection (%) was plotted against compound concentration (µg/ml) to assess antiviral activity..................... 10

Figure S5. a) Dose–response assay curves of H3N2 of C4C11SO4 and C4C11SO3; b) dose–response assay curves of H3N2 of B3C11SO4 and B3C11SO3 on MDCK cells. Viral infection (%) was plotted against compound concentration (µg/ml) to assess antiviral activity. 11

Figure S6. a) Dose–response assay curves of FluB of B3C11SO4 and B3C11SO3; b) dose–response assay curves of FluB of C4C11SO4 and C4C11SO3 on MDCK cells. Viral infection (%) was plotted against compound concentration (µg/ml) to assess antiviral activity. 12

Figure S7. a) Virucidal assay curves of HSV-1 viruses in the presence of C4C11SO4 and C4C11SO3; b) virucidal assay curves of HSV-1 viruses in the presence of B3C6SO4 and B3C6SO3; c) virucidal assay curves of HSV-1 viruses in the presence of B3C11SO4 and B3C11SO3; d) virucidal assay curves of HSV-1 viruses in the presence of C4C3SO3 on Vero cells. Statistical significance was analyzed with two-tailed unpaired t-test. The asterisks represent the p value (ns, 0.1234, ***,0.0002, ****, p<0.0001)... 13

Figure S8. a) Virucidal assay curves of HSV-2 viruses in the presence of C4C11SO3 and C4C11SO4; b) virucidal assay curves of HSV-2 viruses in the presence of B3C6SO4 and B3C6SO3; c) virucidal assay curves of HSV-2 viruses in the presence of B3C11SO4 and B3C11SO3; d) virucidal assay curves of HSV-2 viruses in the presence of C4C3SO3 on Vero cells. Statistical significance was analyzed with two-tailed unpaired t-test. The asterisks represent the p value (ns, 0.1234, ***,0.0002, ****, p<0.0001)... 14

Figure S9. a) Virucidal assay curves of H1N1NO9 viruses in the presence of C4C11SO4 and C4C11SO3; b) virucidal assay curves of H1N1NO9 viruses in the presence of B3C6SO4; c) virucidal assay curves of H1N1NO9 viruses in the presence of B3C11SO4 and B3C11SO3 on MDCK cells. Statistical significance was analyzed with two-tailed unpaired t-test. The asterisks represent the p value (ns, 0.1234, ***,0.0002, ****, p<0.0001). 15

Figure S10. a) Virucidal assay curves of H1N1clinical viruses in the presence of C4C11SO4; b) virucidal assay curves of H1N1clinical viruses in the presence of B3C11SO4 and B3C11SO3. Statistical significance was analyzed with two-tailed unpaired t-test. The asterisks represent the p value (ns, 0.1234, ***,0.0002, ****, p<0.0001). 16

Figure S11. a) Virucidal assay curves of H3N2 viruses in the presence of C4C11SO4 and C4C11SO3; b) virucidal assay curves of H3N2 viruses in the presence of B3C11SO4 and B3C11SO3 on MDCK cells. Statistical significance was analyzed with two-tailed unpaired t-test. The asterisks represent the p value (ns, 0.1234, ***,0.0002, ****, p<0.0001). 17

Figure S12. a) Virucidal assay curves of FluB viruses in the presence of B3C11SO4 and B3C11SO3 b) virucidal assay curves of FluB viruses in the presence of C4S11SO4 and C4C11SO3 on MDCK cells. Statistical significance was analyzed with two-tailed unpaired t-test. The asterisks represent the p value (ns, 0.1234, ***,0.0002, ****, p<0.0001). 18

Figure S13. a) Cytotoxicity assay curves of B3C6SO4 and B3C6SO3; b) cytotoxicity assay curves of B3C11SO4 and B3C11SO3; c) cytotoxicity assay curves of C4C11SO4 and C4C11SO3; d) cytotoxicity assay curves of C4C3SO3 on Vero cells. Cell viability (%) was plotted against compound concentration (µg/ml) to evaluate cytotoxic effects.......... 19

Figure S14. a) Cytotoxicity assay curves of B3C6SO4 and B3C6SO3; b) cytotoxicity assay curves of C4C11SO4 and C4C11SO3; c) cytotoxicity assay curves of B3C11SO4 and B3C11SO3; d) cytotoxicity assay curves of C4C3SO3 on MDCK cells. Cell viability (%) was plotted against compound concentration (µg/ml) to evaluate cytotoxic effects.......... 20

Figure S15. a) Dose–response assay curves of H3N2 of C4C11SO4 and C4C11SO3 in both pre-treatment and post-treatment; b) dose–response assay curves of H3N2 of B3C11SO4 and B3C11SO3 in both pre-treatment and post-treatment on MDCK cells. Viral infection (%) was plotted against compound concentration (µg/ml) to assess antiviral activity. 22

Figure S16. a) Dose–response assay curves of HSV-2 of B3C11SO4 and B3C11SO3 in both pre-treatment and post-treatment; b) dose–response assay curves of HSV-2 of C4C11SO4 and C4C11SO3 in both pre-treatment and post-treatment; c) dose–response assay curves of HSV-2 of B3C6SO4 and B3C6SO3 in both pre-treatment and post-treatment, d) dose–response assay curves of HSV-2 of C4C3SO3 in both pre-treatment and post-treatment on Vero cells. Viral infection (%) was plotted against compound concentration (µg/ml) to assess antiviral activity. 23

Figure S17. ^{13}C-NMR (D2O, 101 MHz) of B3C11SO4.......... 27

Figure S18. ^{1}H-NMR (D2O, 400 MHz) of B3C11SO4.......... 28

Figure S19. ^{13}C-NMR (DMSO-d6, 101 MHz) of B3C11SO3.......... 29

Figure S20. ^{1}H-NMR (DMSO-d6, 400 MHz) of B3C11SO3.......... 30

Figure S21. ^{1}H NMR of (D2O, 400 MHz) compound B3C6SO4. 31

Figure S22. MS (nanochip-ESI/LTQ-orbitrap) of B3C6SO4.......... 32

Figure S23. MS (nanochip-ESI/LTQ-orbitrap) of B3C6SO3.......... 33

Figure S24. ^{1}H-NMR (D2O, 400 MHz) of B3C6SO3.......... 34

Figure S25. ^{1}H NMR (D2O, 400 MHz) of C4C11SO3. 35

Figure S26. MS (nanochip-ESI/LTQ-orbitrap) of C4C11SO3.......... 36

Figure S27. ^{1}H NMR (D2O, 400 MHz) of C4C11SO4. 37

Figure S28. MS (nanochip-ESI/LTQ-orbitrap) of C4C11SO4.......... 38

Figure S29. ^{1}H NMR (D2O, 400 MHz) of C4C3SO3. 39

Figure S30. MS (nanochip-ESI/LTQ-orbitrap) of C4C3SO3. 40

Figure S31. ESI-MS spectra of compound B3C11SO4. 41

Tables

Table S1. The cytotoxicity results of MEIs on Vero cells and MDCK cells. 21

Table S2. The HSV-2 virucidal results of MEIs at the different concentrations. 24

Table S3. The K_d values of interactions between MEIs and BSA in water. 25

Table S4. The K_d values of interactions between MEIs and viral proteins at 25°C. 26

1. Supplementary figures and tables

1.1. Viral inhibition assays performed with MEIs

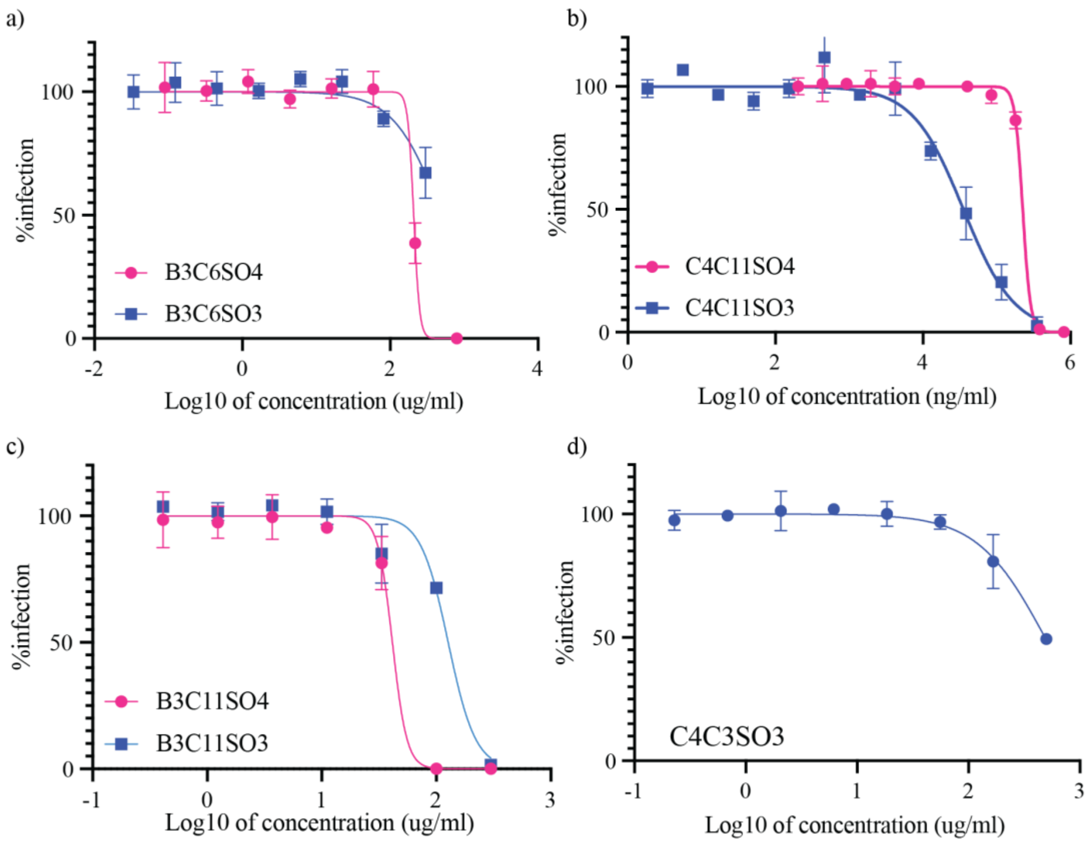

Figure S1. (a)Dose–response assay curves of HSV-1 in the presence of B3C6SO4 and B3C6SO3; (b) dose–response assay curves of HSV-1 in the presence of C4C11SO4 and C4C11SO3, c) dose–response assay curves of HSV-1 in the presence of B3C11SO4 and B3C11SO3; d) dose–response assay curves of HSV-1 in the presence of C4C3SO3 on Vero cells. Viral infection (%) was plotted against compound concentration (µg/ml) to assess antiviral activity.

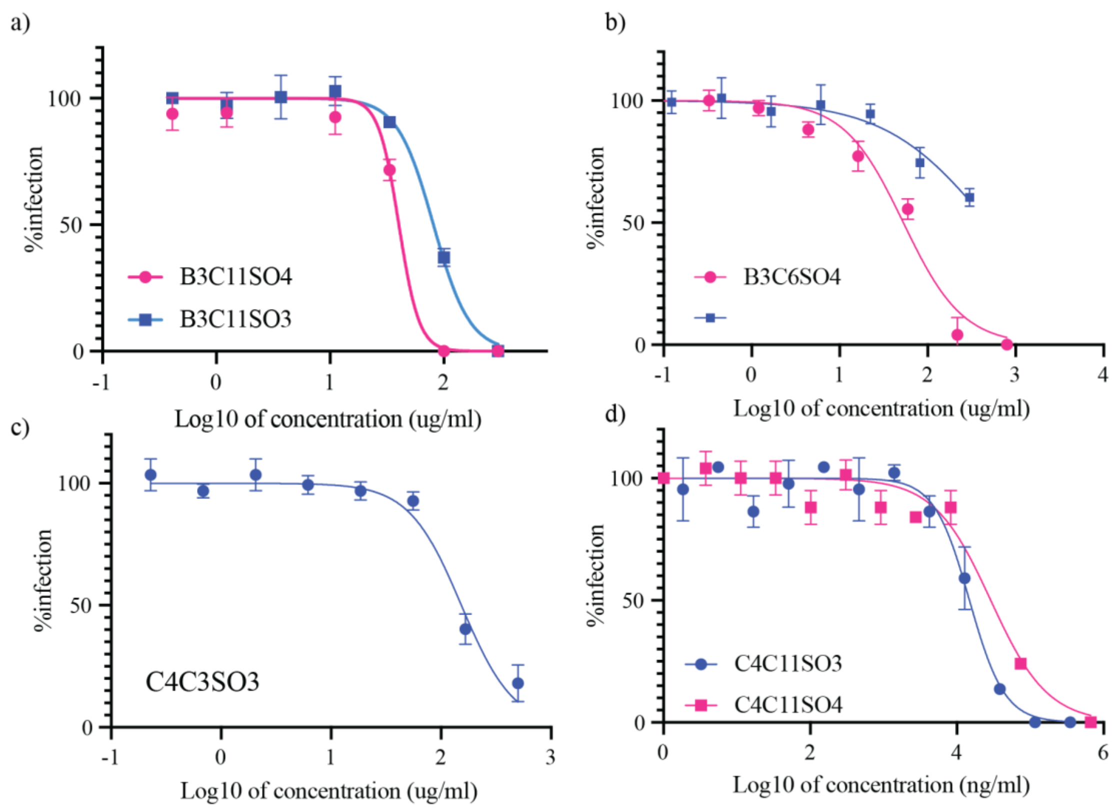

Figure S2. a) Dose–response assay curves of HSV-2 in the presence of B3C11SO4 and B3C11SO3; b) dose–response assay curves of HSV-2 in the presence of B3C6SO4 and B3C6SO4; c) dose–response assay curves of HSV-2 in the presence of C4C3SO3; d) dose–response assay curves of HSV-2 in the presence of C4C11SO4 and C4C11SO3 on Vero cells. Viral infection (%) was plotted against compound concentration (µg/ml) to assess antiviral activity.

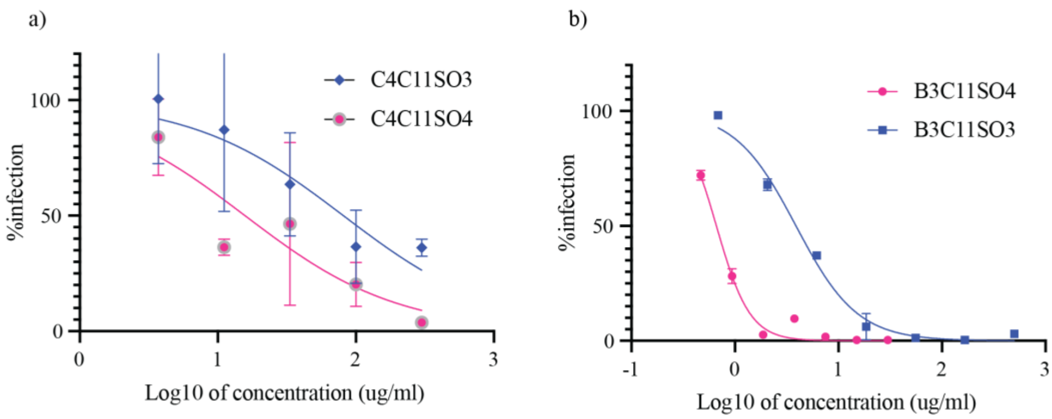

Figure S3. a) Dose–response assay curves of H1N1 Clinical of C4C11SO4 and C4C11SO3; b) dose–response assay curves of H1N1 Clinical of B3C11SO4 and B3C11SO3 on MDCK cells. Viral infection (%) was plotted against compound concentration (µg/ml) to assess antiviral activity.

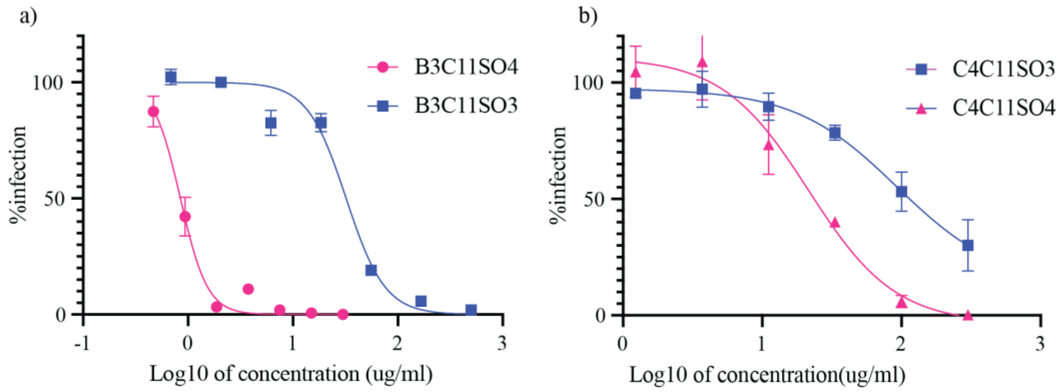

Figure S4. a) Dose–response assay curves of H1N1 NO9 of B3C11SO3 and B3C11SO4; b) dose–response assay curves of H1N1 NO9 of C4C11SO3 and C4C11SO4 on MDCK cells. Viral infection (%) was plotted against compound concentration (μg/ml) to assess antiviral activity.

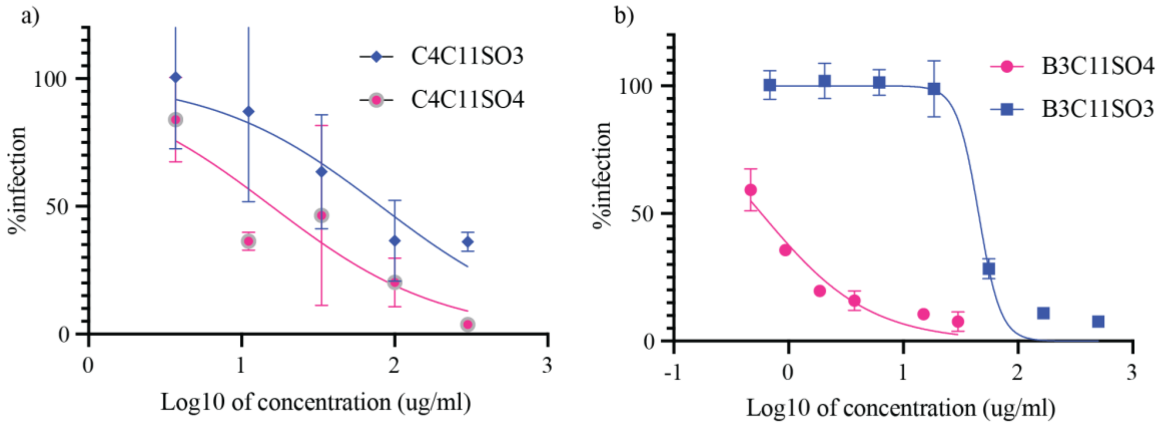

Figure S5. a) Dose–response assay curves of H3N2 of C4C11SO4 and C4C11SO3; b) dose–response assay curves of H3N2 of B3C11SO4 and B3C11SO3 on MDCK cells. Viral infection (%) was plotted against compound concentration (µg/ml) to assess antiviral activity.

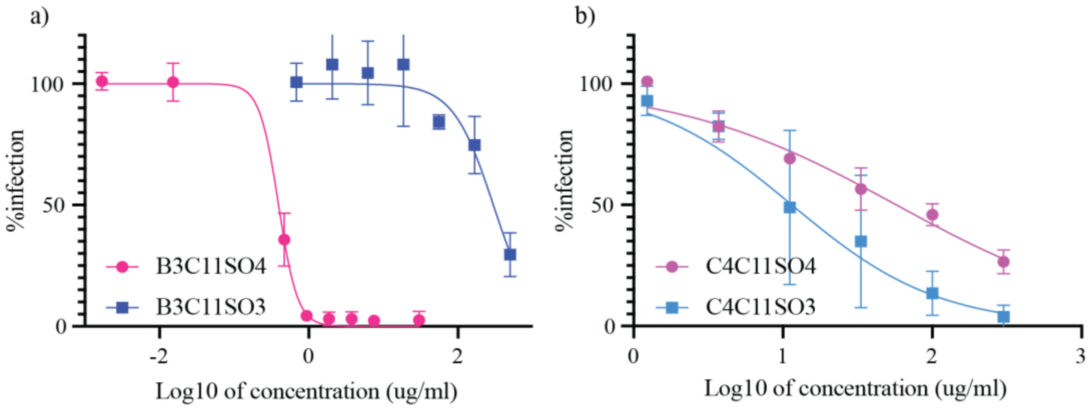

Figure S6. a) Dose–response assay curves of FluB of B3C11SO4 and B3C11SO3; b) dose–response assay curves of FluB of C4C11SO4 and C4C11SO3 on MDCK cells. Viral infection (%) was plotted against compound concentration (µg/ml) to assess antiviral activity.

1.2.The virucidal assays

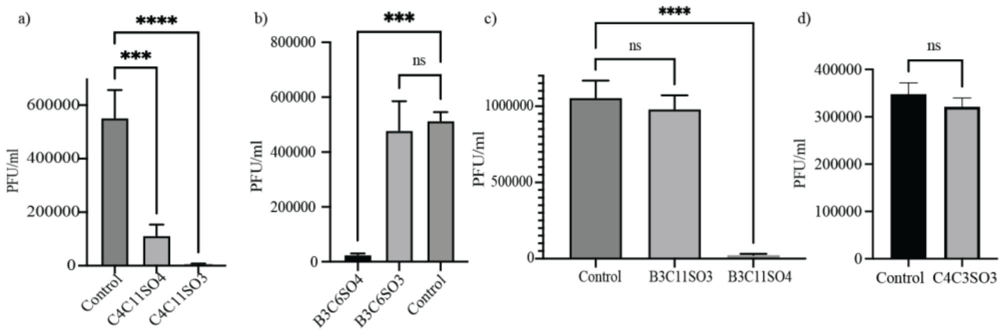

Figure S7. a) Virucidal assay curves of HSV-1 viruses in the presence of C4C11SO4 and C4C11SO3; b) virucidal assay curves of HSV-1 viruses in the presence of B3C6SO4 and B3C6SO3; c) virucidal assay curves of HSV-1 viruses in the presence of B3C11SO4 and B3C11SO3; d) virucidal assay curves of HSV-1 viruses in the presence of C4C3SO3 on Vero cells. Statistical significance was analyzed with two-tailed unpaired t-test. The asterisks represent the p value (ns, 0.1234, ***,0.0002, ****, p<0.0001).

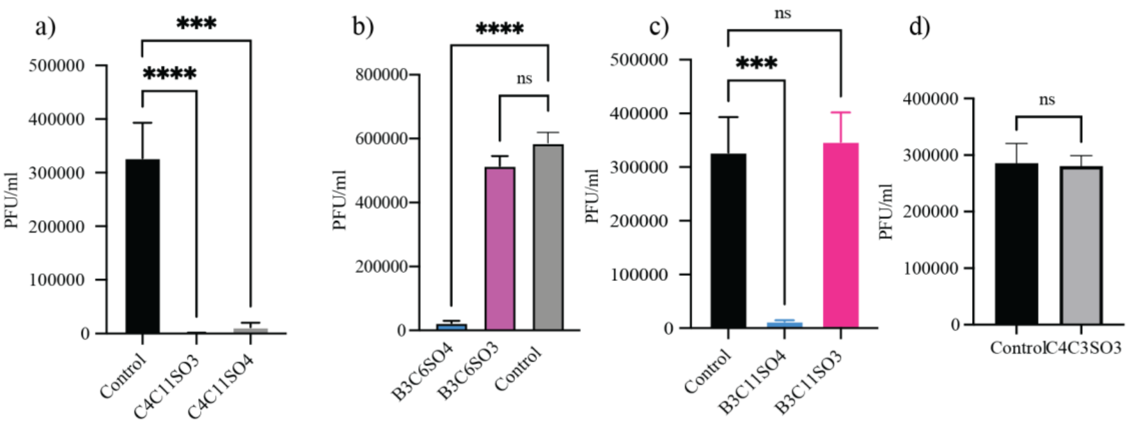

Figure S8. a) Virucidal assay curves of HSV-2 viruses in the presence of C4C11SO3 and C4C11SO4; b) virucidal assay curves of HSV-2 viruses in the presence of B3C6SO4 and B3C6SO3; c) virucidal assay curves of HSV-2 viruses in the presence of B3C11SO4 and B3C11SO3; d) virucidal assay curves of HSV-2 viruses in the presence of C4C3SO3 on Vero cells. Statistical significance was analyzed with two-tailed unpaired t-test. The asterisks represent the p value (ns, 0.1234, ***,0.0002, ****, p<0.0001).

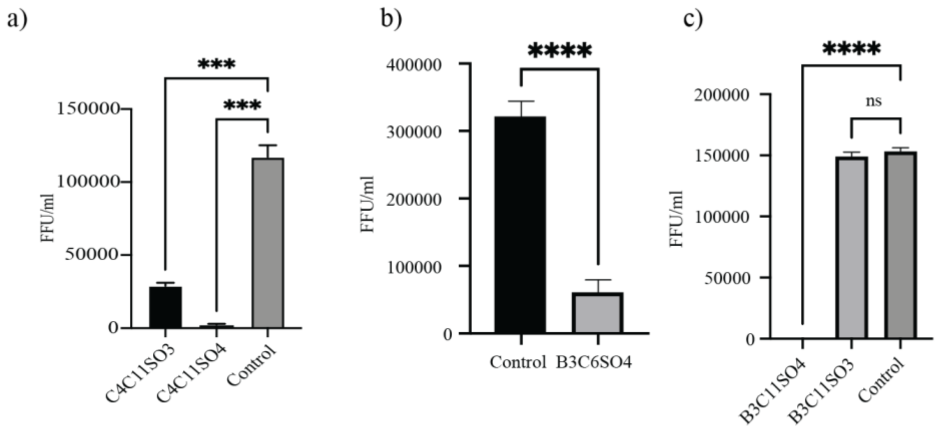

Figure S9. a) Virucidal assay curves of H1N1NO9 viruses in the presence of C4C11SO4 and C4C11SO3; b) virucidal assay curves of H1N1NO9 viruses in the presence of B3C6SO4; c) virucidal assay curves of H1N1NO9 viruses in the presence of B3C11SO4 and B3C11SO3 on MDCK cells. Statistical significance was analyzed with two-tailed unpaired t-test. The asterisks represent the p value (ns, 0.1234, ***,0.0002, ****, $p<0.0001$).

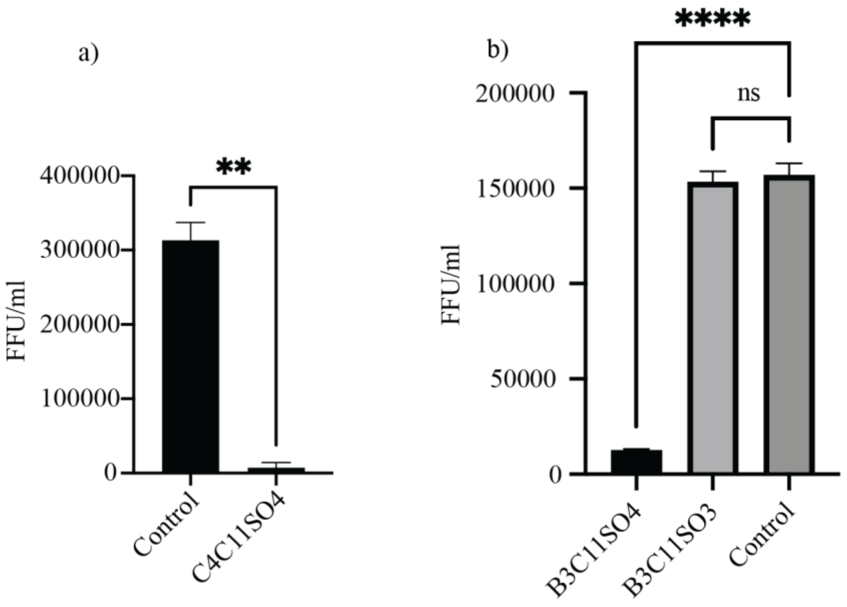

Figure S10. a) Virucidal assay curves of H1N1clinical viruses in the presence of C4C11SO4; b) virucidal assay curves of H1N1clinical viruses in the presence of B3C11SO4 and B3C11SO3. Statistical significance was analyzed with two-tailed unpaired t-test. The asterisks represent the p value (ns, 0.1234, ***,0.0002, ****, p<0.0001).

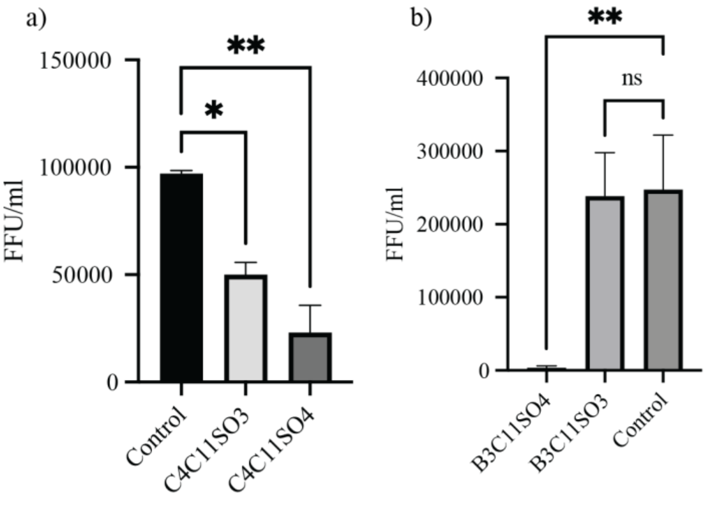

Figure S11. a) Virucidal assay curves of H3N2 viruses in the presence of C4C11SO4 and C4C11SO3; b) virucidal assay curves of H3N2 viruses in the presence of B3C11SO4 and B3C11SO3 on MDCK cells. Statistical significance was analyzed with two-tailed unpaired t-test. The asterisks represent the p value (ns, 0.1234, ***,0.0002, ****, p<0.0001).

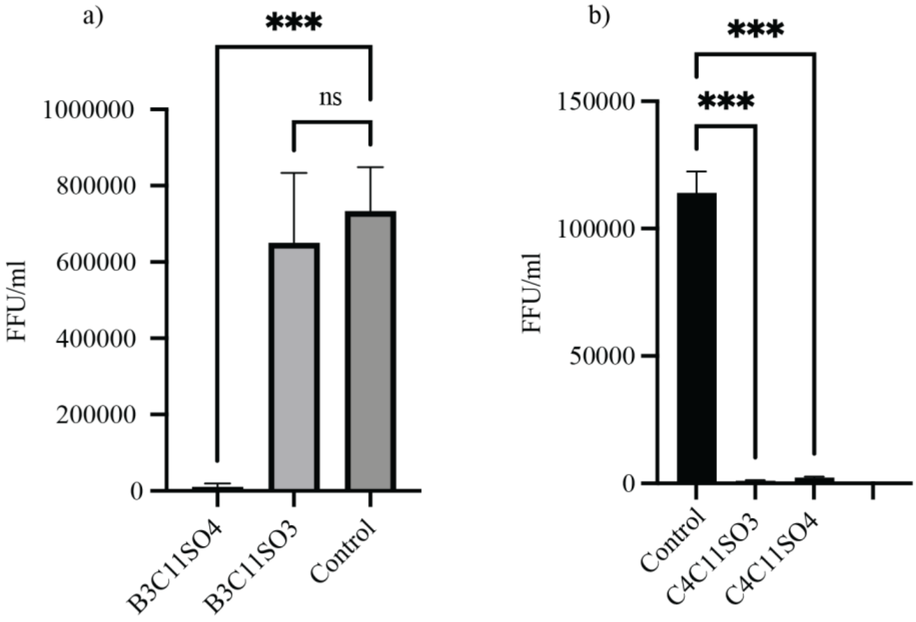

Figure S12. a) Virucidal assay curves of FluB viruses in the presence of B3C11SO4 and B3C11SO3 b) virucidal assay curves of FluB viruses in the presence of C4S11SO4 and C4C11SO3 on MDCK cells. Statistical significance was analyzed with two-tailed unpaired t-test. The asterisks represent the p value (ns, 0.1234, ***,0.0002, ****, p<0.0001).

1.3.The cytotoxicity assays performed with MEIs

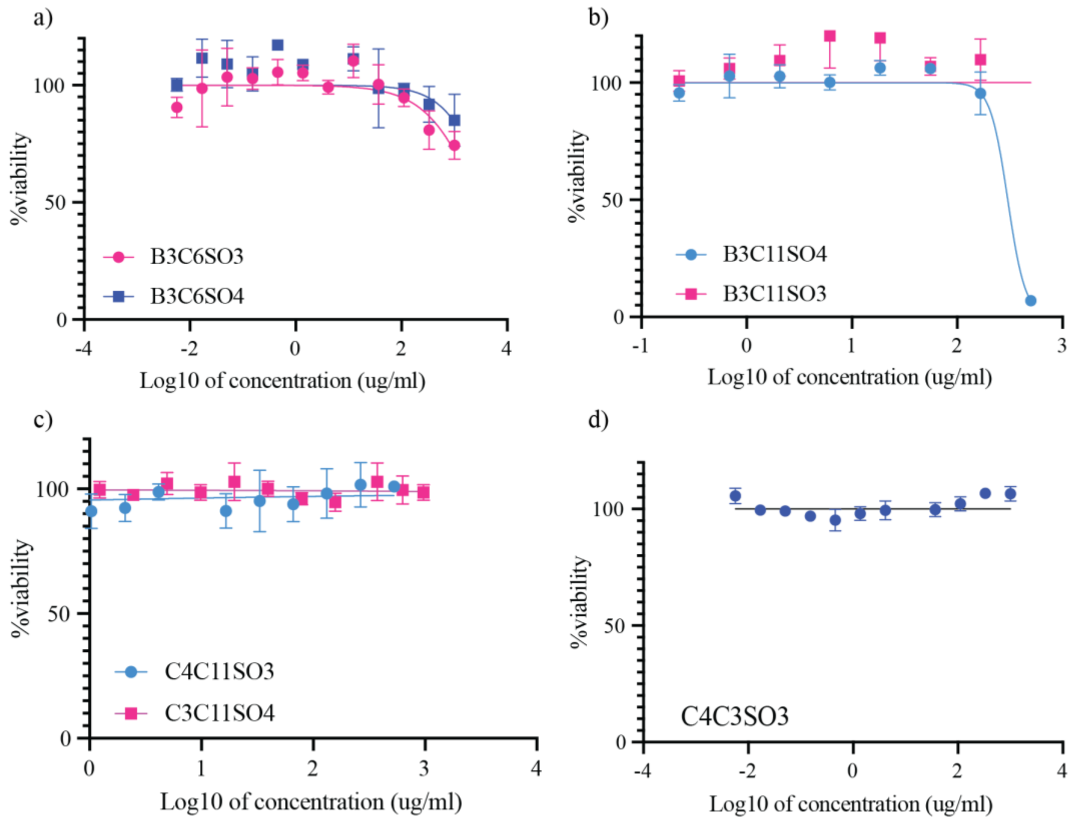

Figure S13. a) Cytotoxicity assay curves of B3C6SO4 and B3C6SO3; b) cytotoxicity assay curves of B3C11SO4 and B3C11SO3; c) cytotoxicity assay curves of C4C11SO4 and C4C11SO3; d) cytotoxicity assay curves of C4C3SO3 on Vero cells. Cell viability (%) was plotted against compound concentration (µg/ml) to evaluate cytotoxic effects.

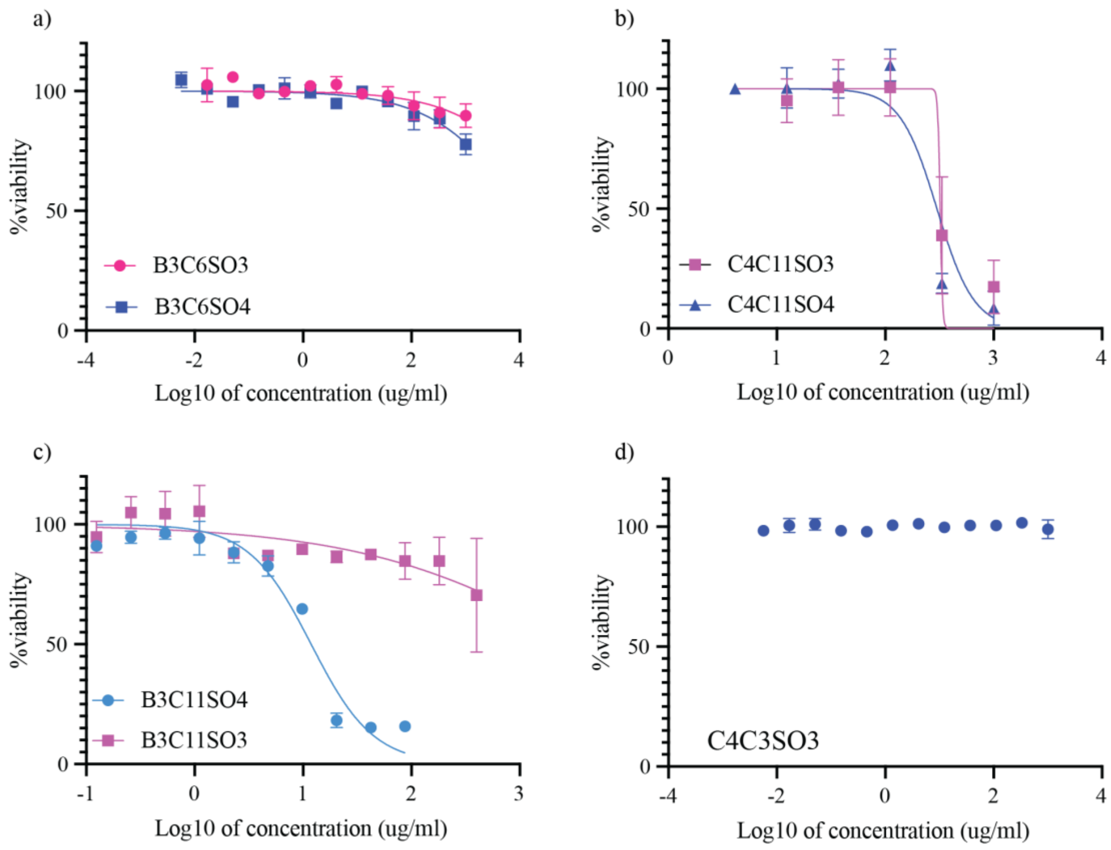

Figure S14. a) Cytotoxicity assay curves of B3C6SO4 and B3C6SO3; b) cytotoxicity assay curves of C4C11SO4 and C4C11SO3; c) cytotoxicity assay curves of B3C11SO4 and B3C11SO3; d) cytotoxicity assay curves of C4C3SO3 on MDCK cells. Cell viability (%) was plotted against compound concentration (µg/ml)

Table S1. The cytotoxicity results of MEIs on Vero cells and MDCK cells.

Compounds	CC_{50}(μM)	
	Vero cells	MDCK cells
B3C11SO4	313.38±28.73	12.25±0.94
B3C11S03	>546	>415.34
B3C6SO4	>1000	>1000
B3C6SO3	>1000	>1000
C4C11SO4	>657.29	>194
C4C11SO3	>358.1	202.27±0.76
C4C3SO4	>1000	>1000
C4C3SO3	>1000	>1000

1.4.The inhibition assays of MEIs in the growth of viruses

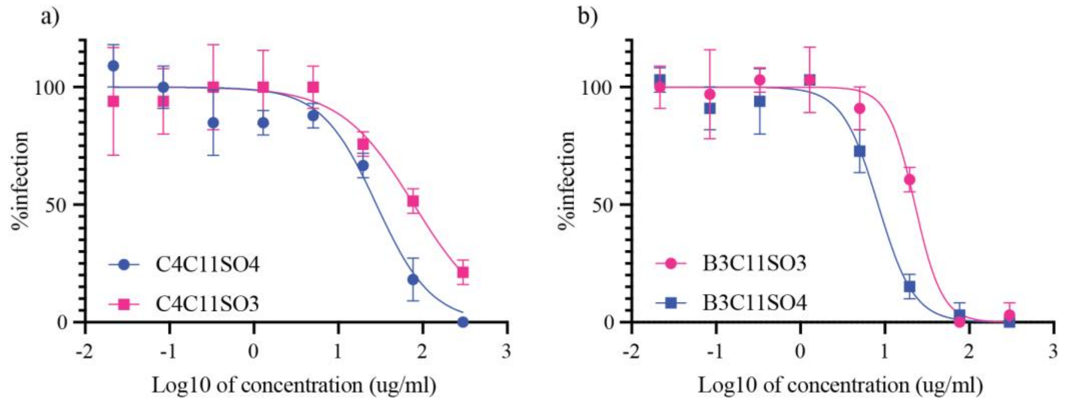

Figure S15. a) Dose–response assay curves of H3N2 of C4C11SO4 and C4C11SO3 in both pre-treatment and post-treatment; b) dose–response assay curves of H3N2 of B3C11SO4 and B3C11SO3 in both pre-treatment and post-treatment on MDCK cells. Viral infection (%) was plotted against compound concentration (µg/ml) to assess antiviral activity.

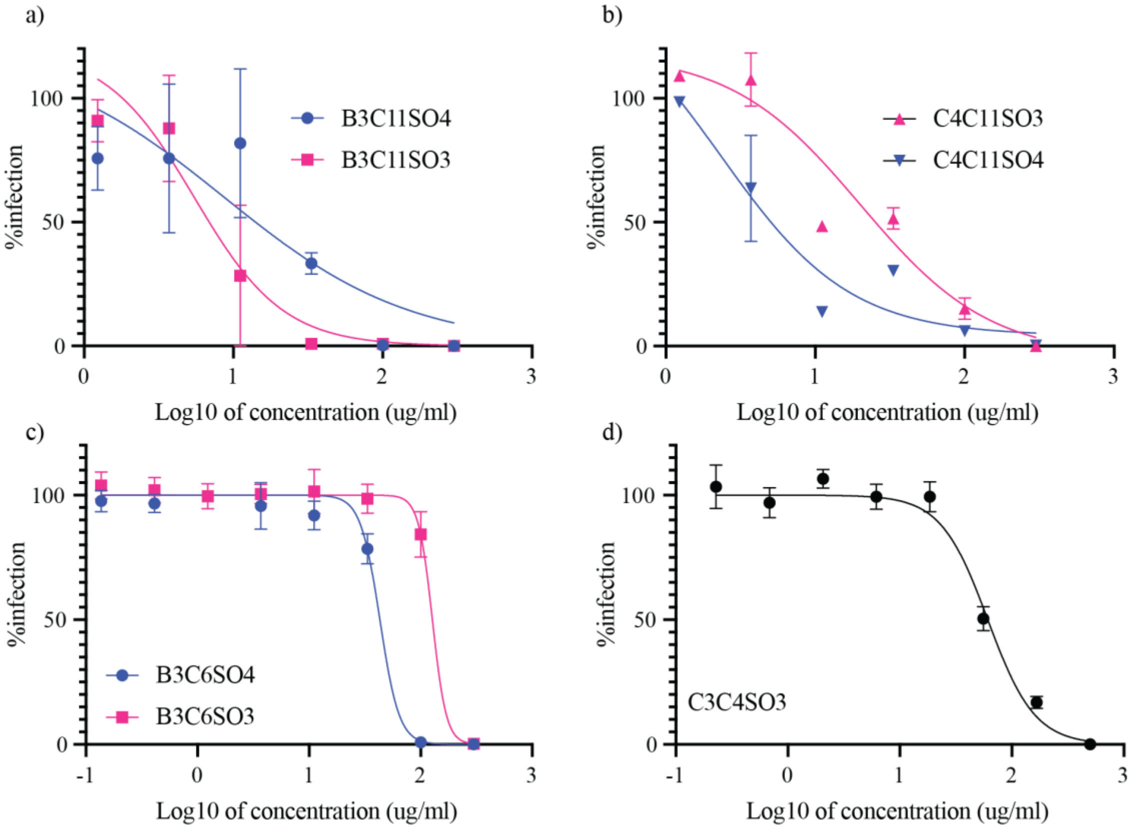

Figure S16. a) Dose–response assay curves of HSV-2 of B3C11SO4 and B3C11SO3 in both pre-treatment and post-treatment; b) dose–response assay curves of HSV-2 of C4C11SO4 and C4C11SO3 in both pre-treatment and post-treatment; c) dose–response assay curves of HSV-2 of B3C6SO4 and B3C6SO3 in both pre-treatment and post-treatment, d) dose–response assay curves of HSV-2 of C4C3SO3 in both pre-treatment and post-treatment on Vero cells. Viral infection (%) was plotted against compound concentration (µg/ml) to assess antiviral activity.

1.5.The relationship between CMC and virucidal properties

Table S2. The HSV-2 virucidal results of MEIs at the different concentrations.

Compounds	Concentration (uM)	Virucidal/Virustatic
C4C11SO3	304	Virucidal
	237.16	Virucidal
	126.3	Virucidal
B3C11SO3	300	Virustatic
	500	Virustatic

1.6. Dissociation constant of interactions between MEIs and BSA protein

Table S3. The K_d values of interactions between MEIs and BSA in water.

Compounds	Buffers	K_d (μM)	ΔH (kcal/mol)	ΔG (kcal/mol)	-ΔTS (kcal/mol)	N	Experimental temperature °C
B3C11SO4	PBS	3.8	2.46	-7.46	-9.86	3.21	25
		1.37	1.48	-8.32	-9.8	2.58	37
B3C11SO3	H_2O	5.82	-9.08	-7.15	1.93	1.1	25
		4.97	-9.78	-7.53	0.272	0.838	37
B3C6SO4	H_2O	7.27	-14.5	-7.29	7.2	1.69	25
		1.4	-8.93	-7.99	0.938	1.3	37
B3C6SO3	H_2O	No binding					
C4C11SO4	PBS	161	-1.58	-5.18	-3.6	10	25
		8.47	-2.49	-7.2	-4.71	7.6	37
C4C11SO3	PBS	124	-1.58	-5.33	-2.06	10	25
		1.64	-0.315	-8.21	-7.9	2.6	37
C4C3SO3	H_2O	63	-7.34	-5.73	1.61	0.5	25
		52.1	-30.6	-6.08	24.6	0.397	37

1.7. Dissociation constant of interactions between MEIs and viral proteins

Table S4. The K_d values of interactions between MEIs and viral proteins at 25°C.

Proteins	Compounds	K_d (uM)	ΔH (kcal/mol)	ΔG (kcal/mol)	-ΔTS (kcal/mol)
HA1	B3C11SO4	16.9	-12.3	-6.51	5.81
	B3C11SO3	17.1	-6.97	-6.5	0.462
	B3C6SO4	26.6	-6.23	-6.24	-0.012
	B3C6SO3	No binding			
	C4C11SO4	23.3	-6.7	-6.32	0.385
	C4C11SO3	29.1	-5.97	-6.19	-0.223
Glycoprotein D	B3C11SO4	28.2	-2.04	-6.21	-4.17
	B3C11SO3	23.2	-1.28	-6.32	-5.04
	B3C6SO4	28.1	-1.6	-6.21	-4.61
	B3C6SO3	14.2	-3.02	-6.61	-3.59
	C4C11SO4	141	-0.875	-5.25	-4.38
	C4C11SO3	83.5	-0.839	-5.56	-4.72

2. The results of synthesis

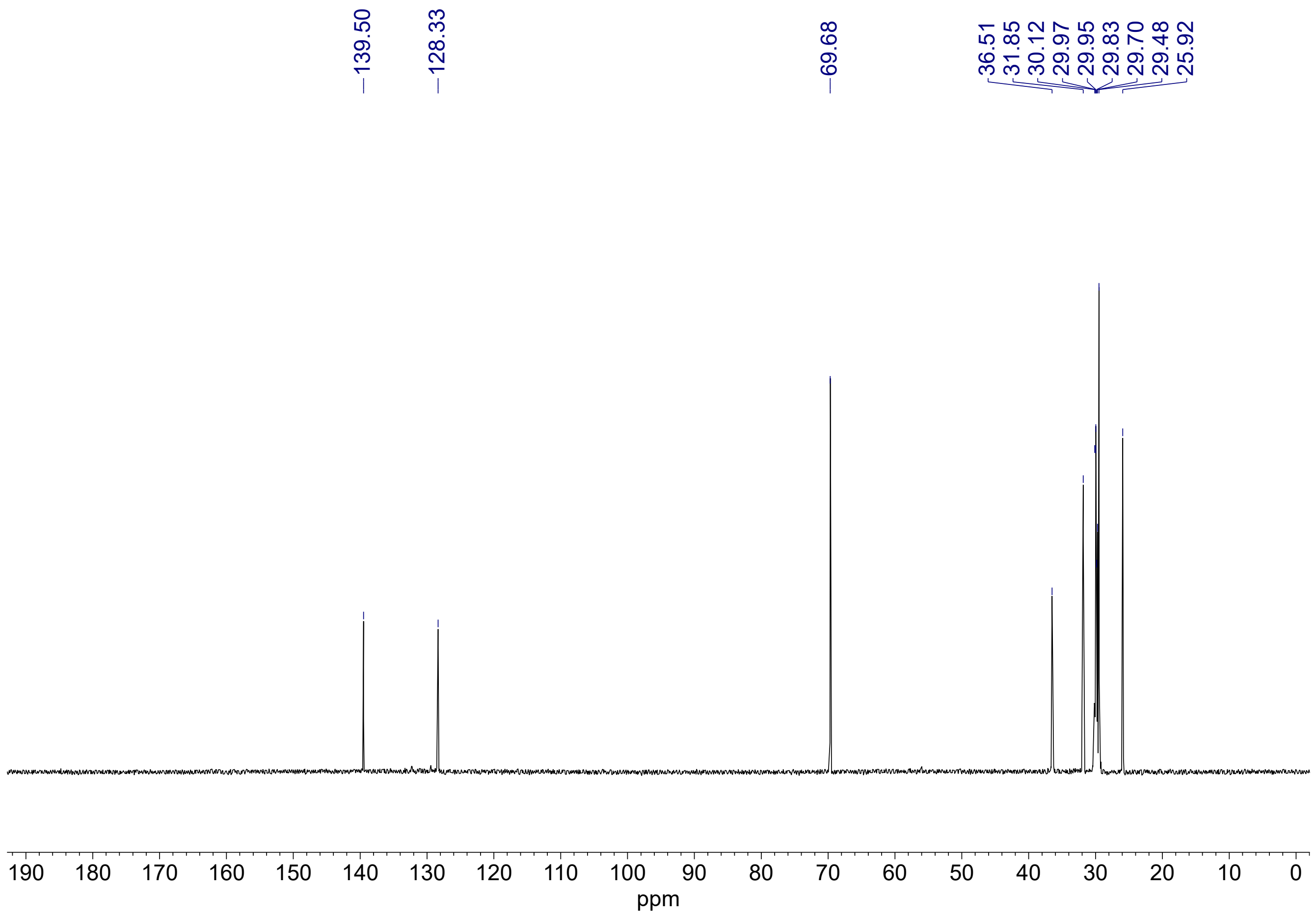

Figure S17. ^{13}C-NMR (D2O, 101 MHz) of B3C11SO4.

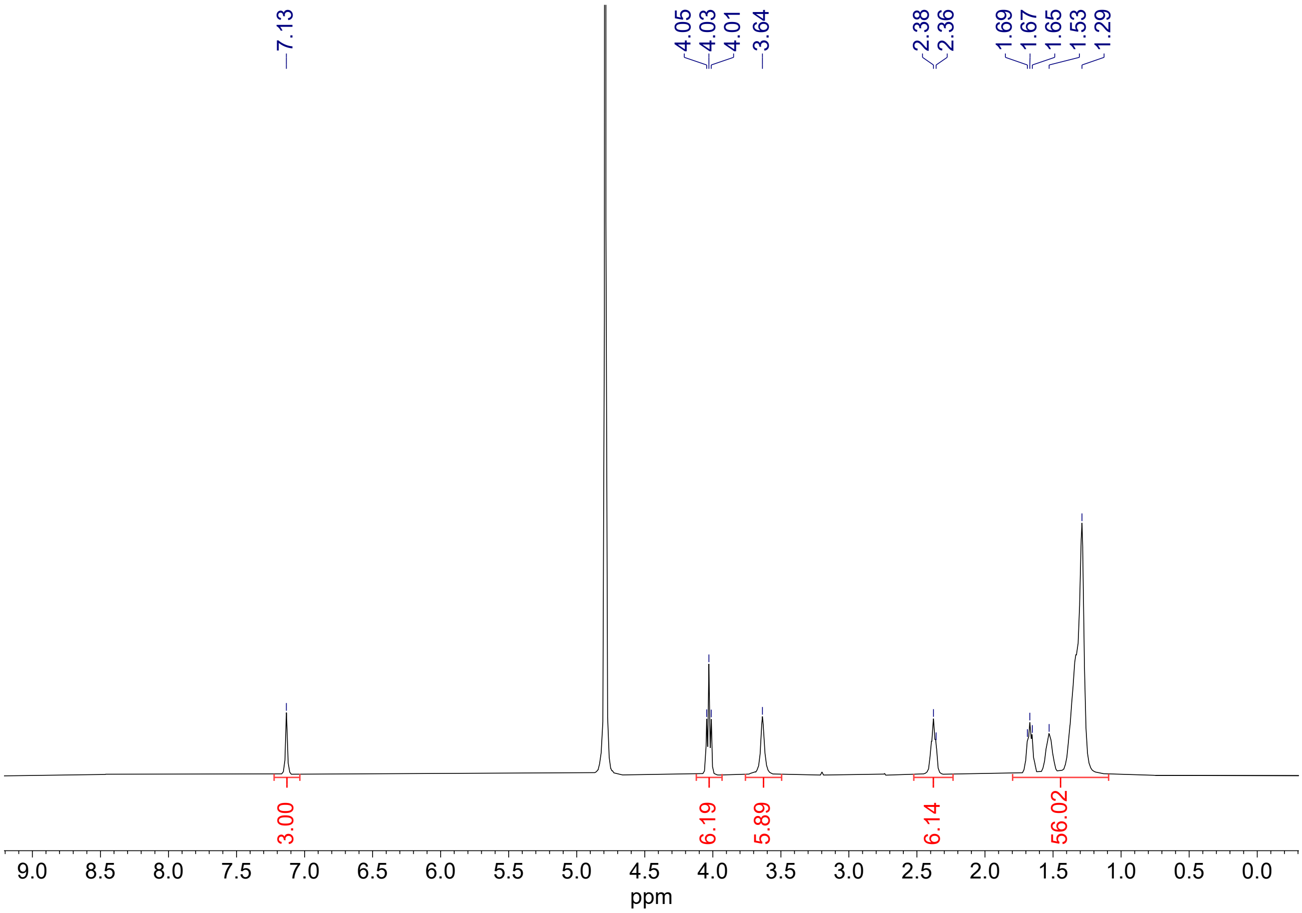

Figure S18. ^{1}H-NMR (D2O, 400 MHz) of B3C11SO4.

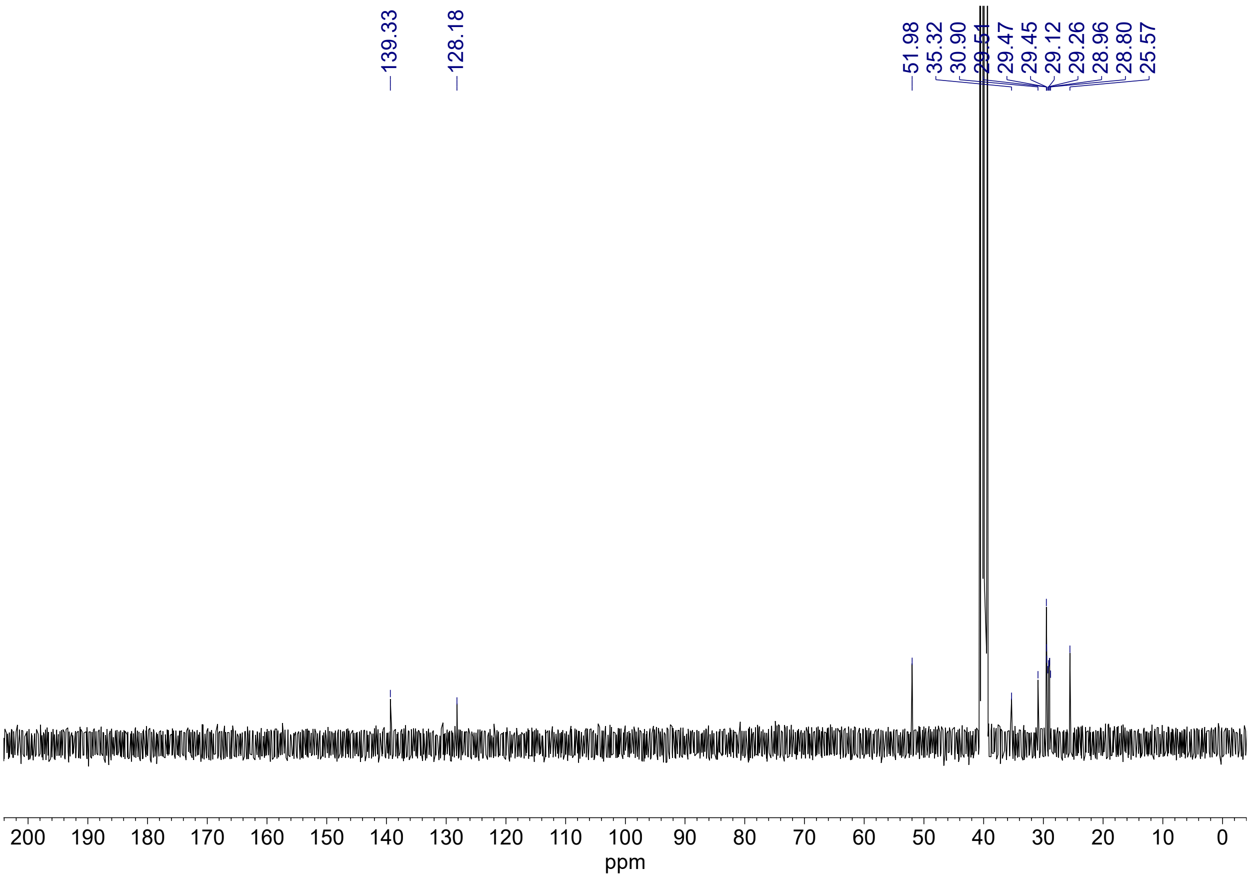

Figure S19. ^{13}C-NMR (DMSO-d6, 101 MHz) of B3C11SO3.

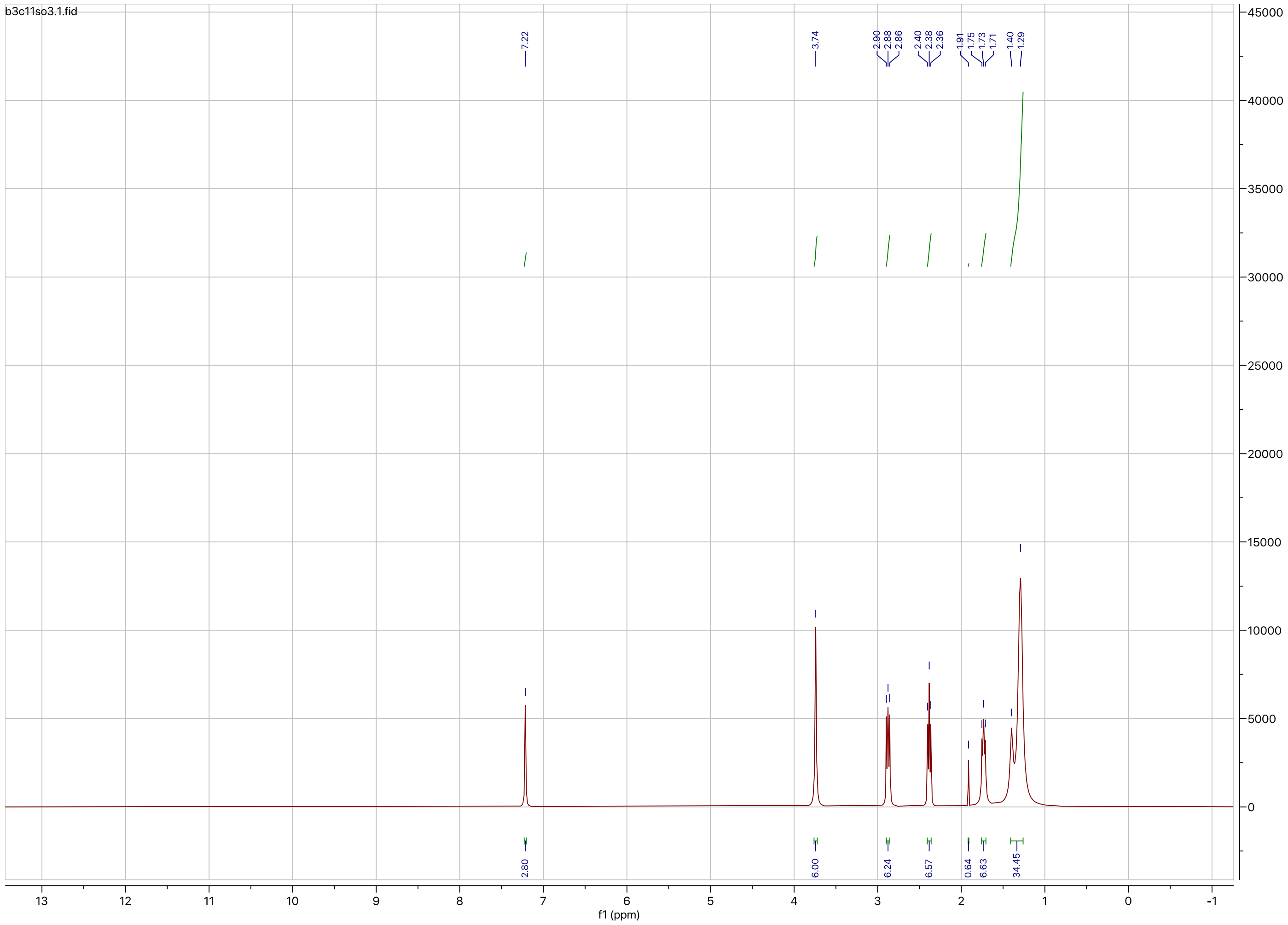

Figure S20. ^{1}H-NMR (DMSO-d6, 400 MHz) of B3C11SO3.

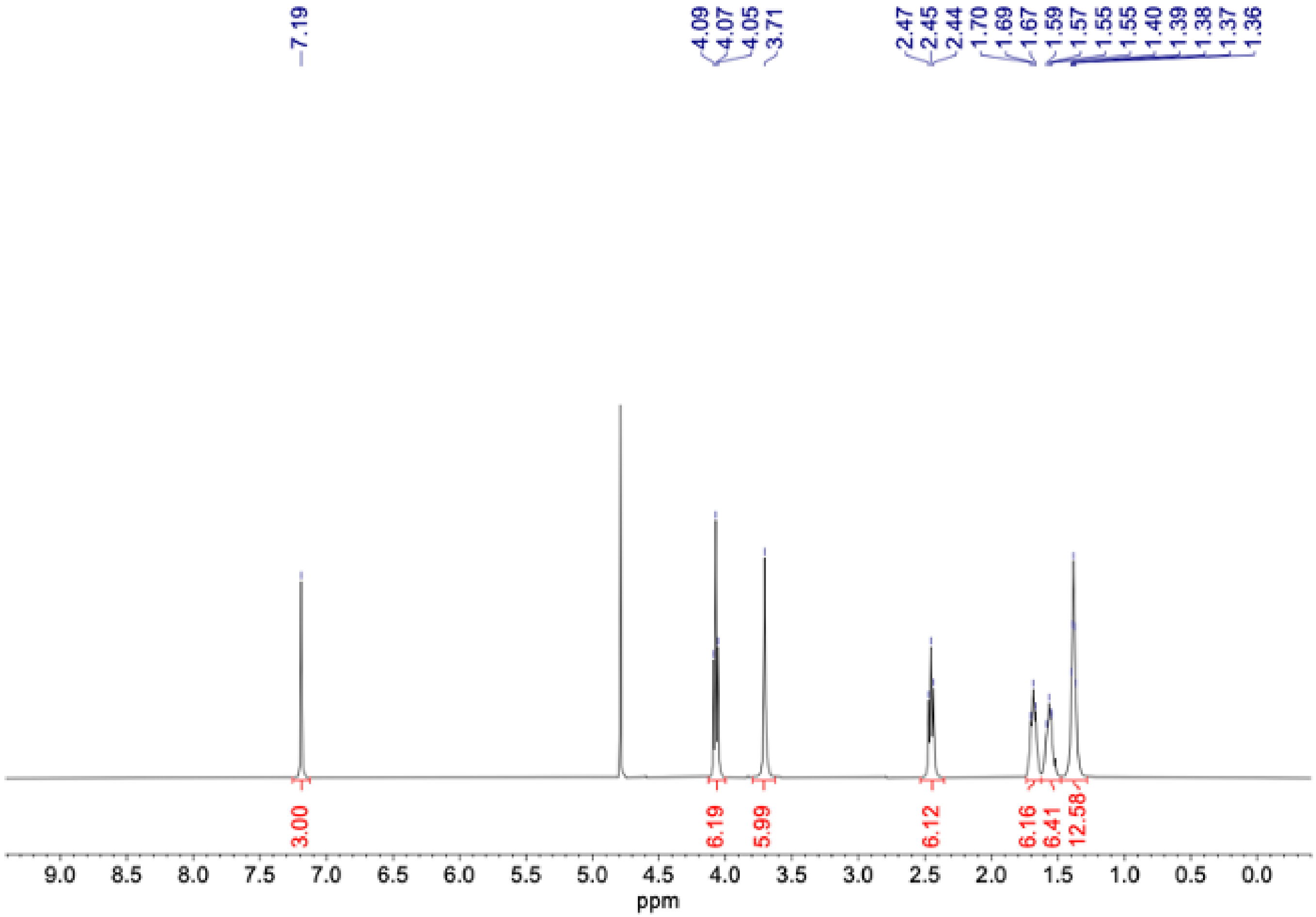

Figure S21. ^{1}H NMR of (D2O, 400 MHz) compound B3C6SO4.

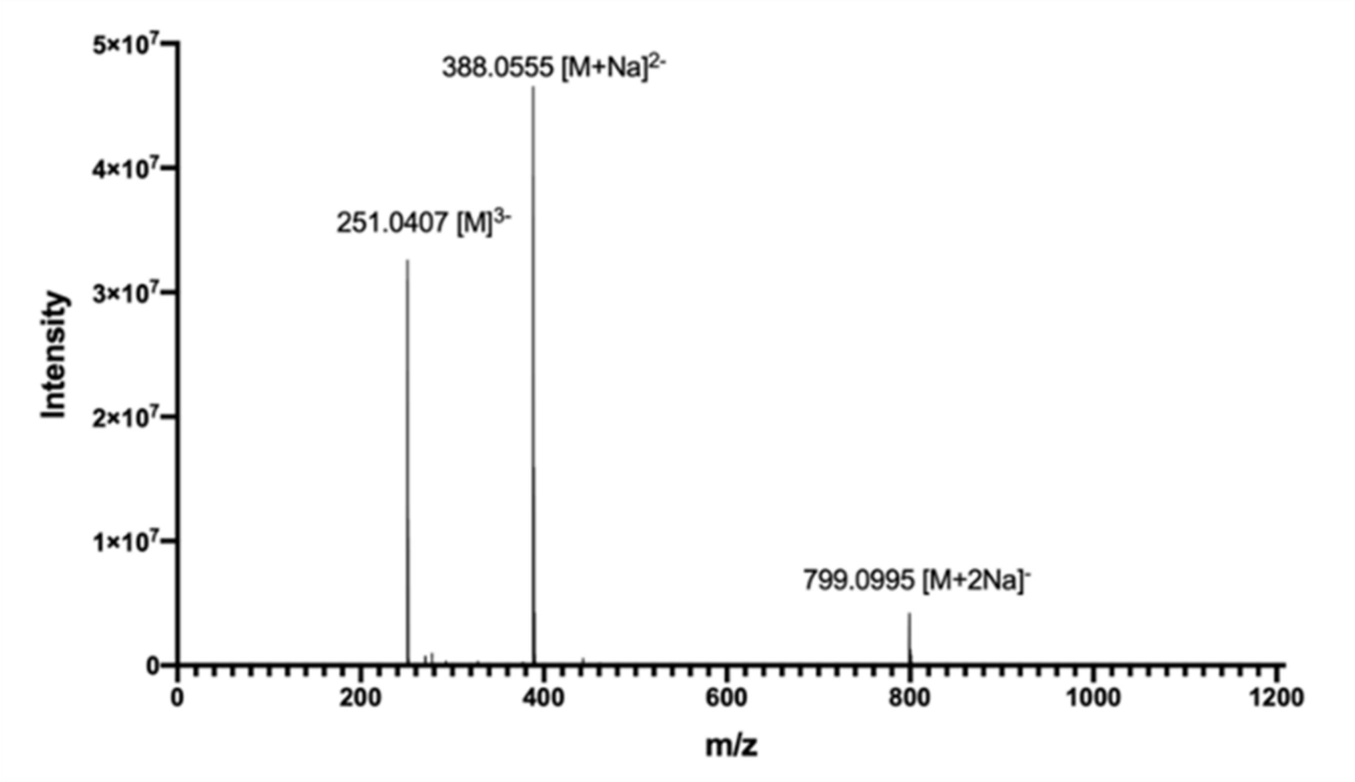

Figure S22. MS (nanochip-ESI/LTQ-orbitrap) of B3C6SO4.

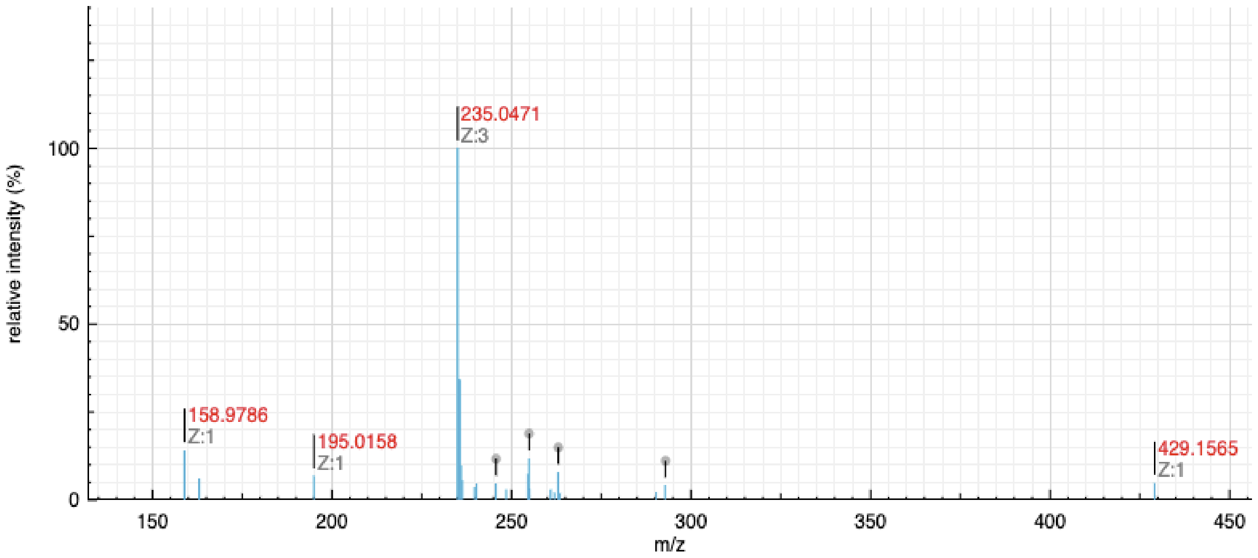

Figure S23. MS (nanochip-ESI/LTQ-orbitrap) of B3C6SO3.

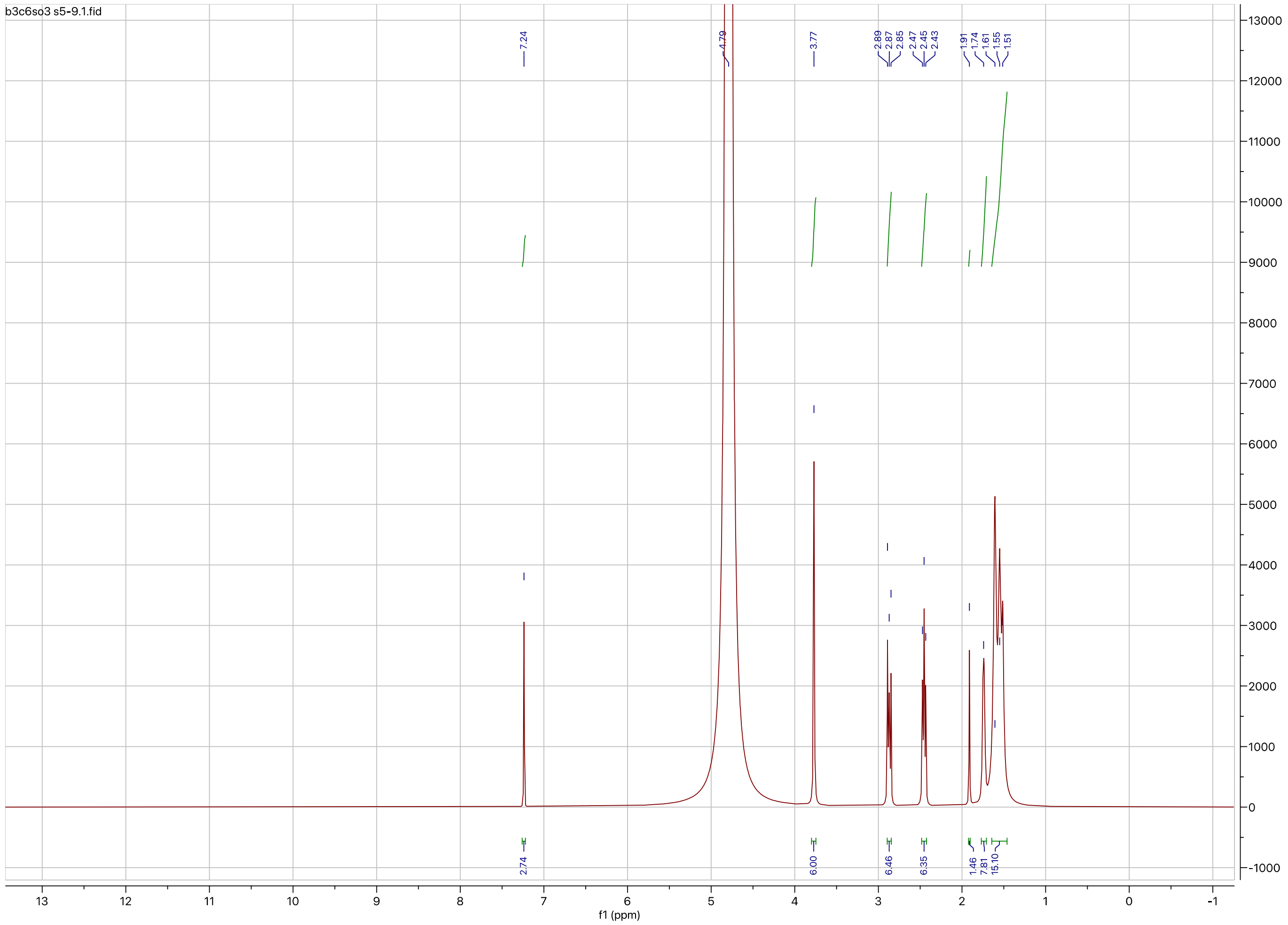

Figure S24. ^{1}H-NMR (D2O, 400 MHz) of B3C6SO3.

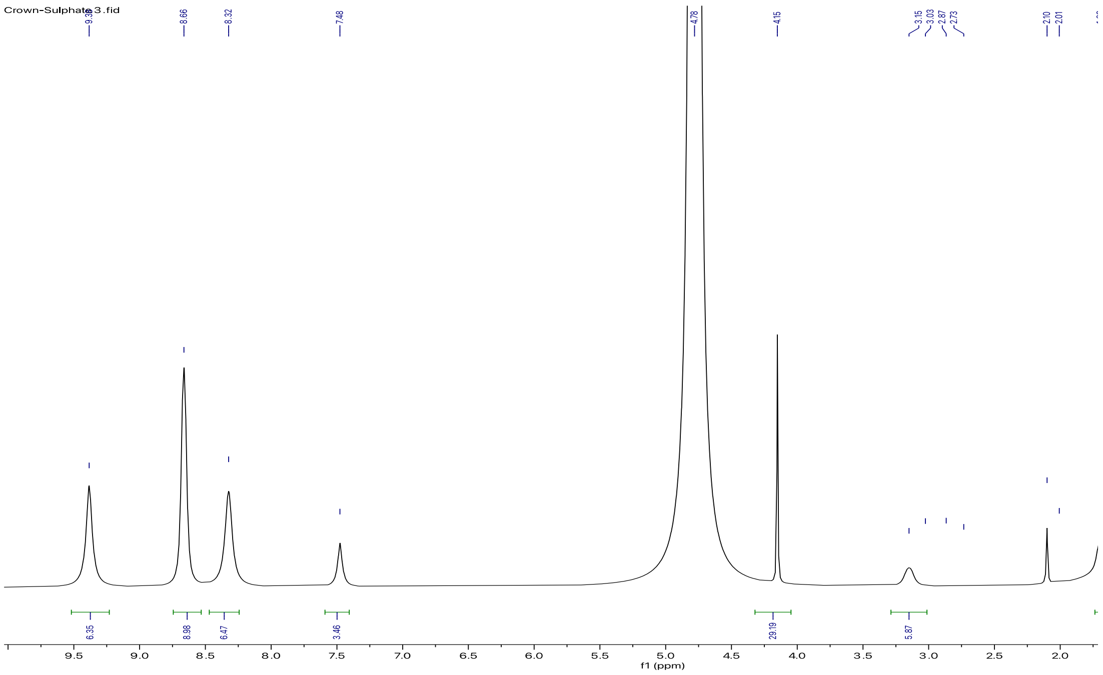

Figure S25. ^{1}H NMR (D2O, 400 MHz) of C4C11SO3.

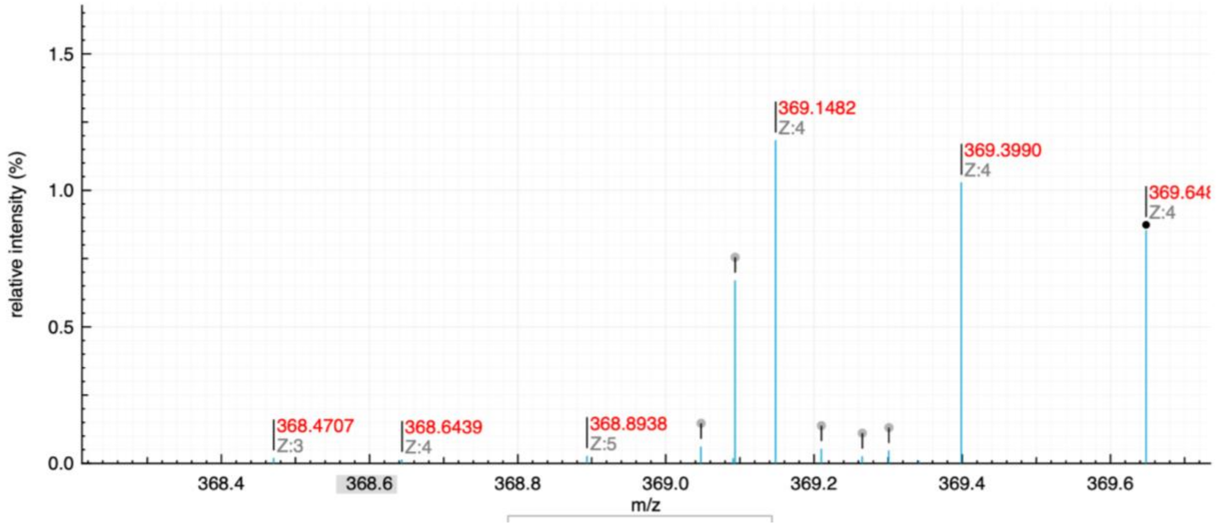

Figure S26. MS (nanochip-ESI/LTQ-orbitrap) of C4C11SO3.

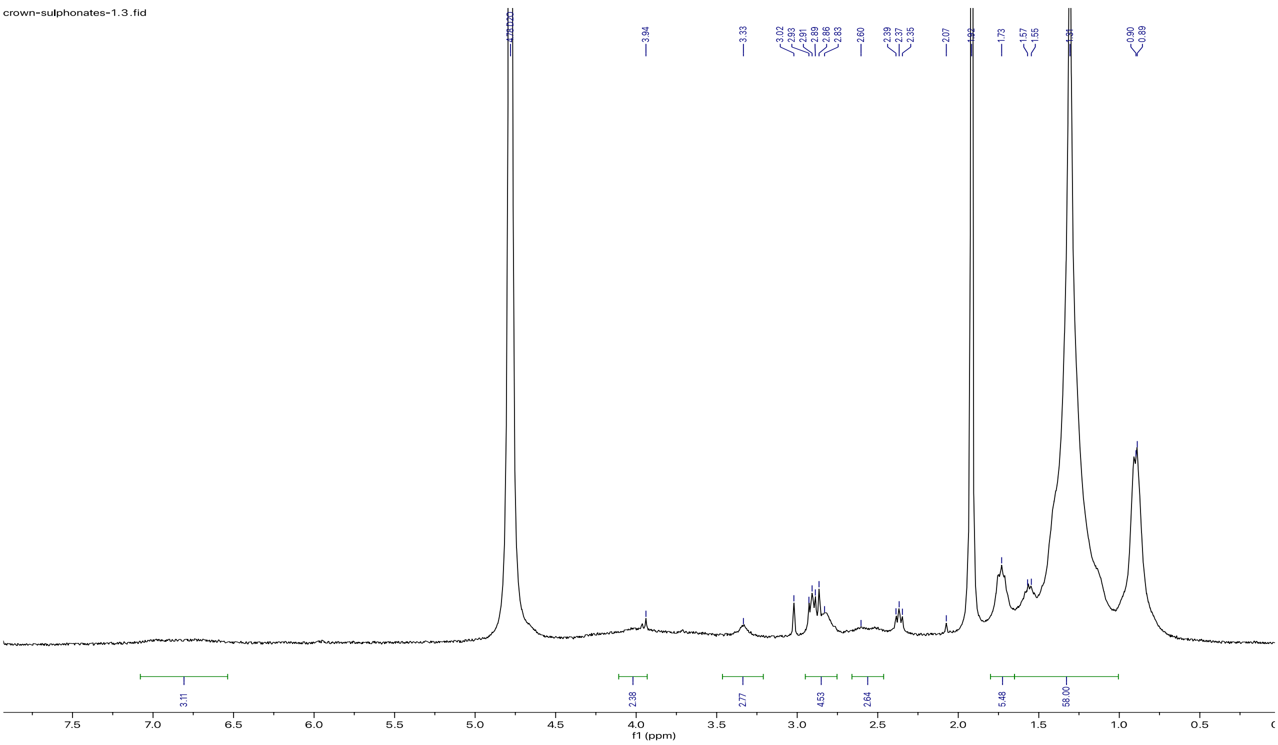

Figure S27. ^{1}H NMR (D2O, 400 MHz) of C4C11SO4.

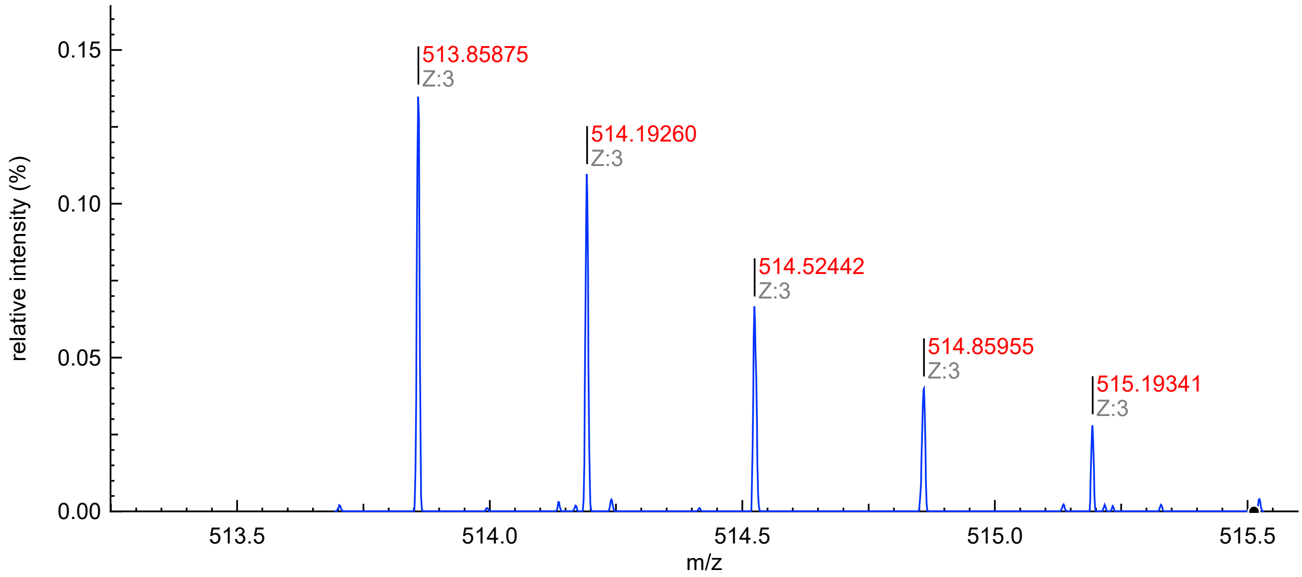

Figure S28. MS (nanochip-ESI/LTQ-orbitrap) of C4C11SO4.

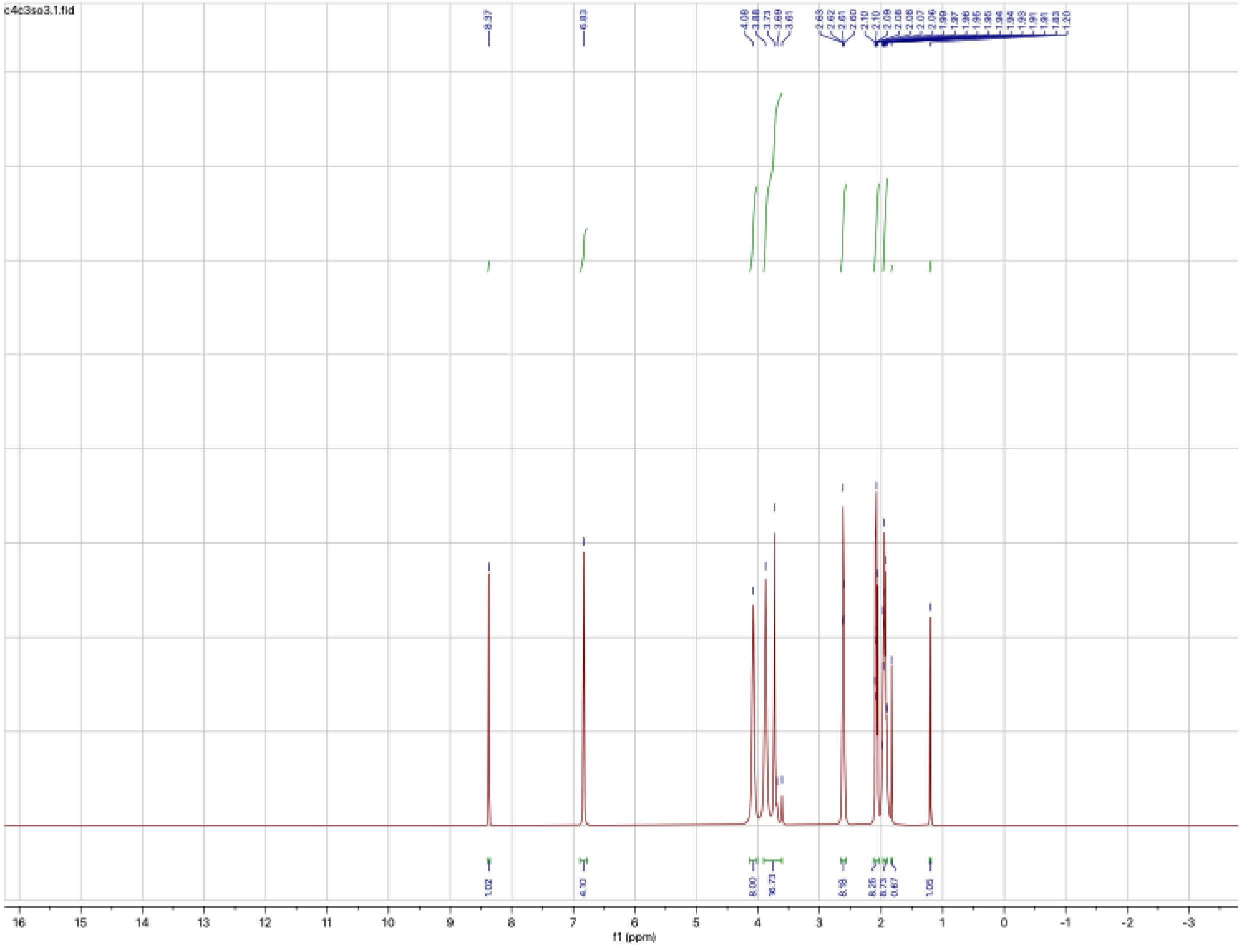

Figure S29. ^{1}H NMR (D2O, 400 MHz) of C4C3SO3.

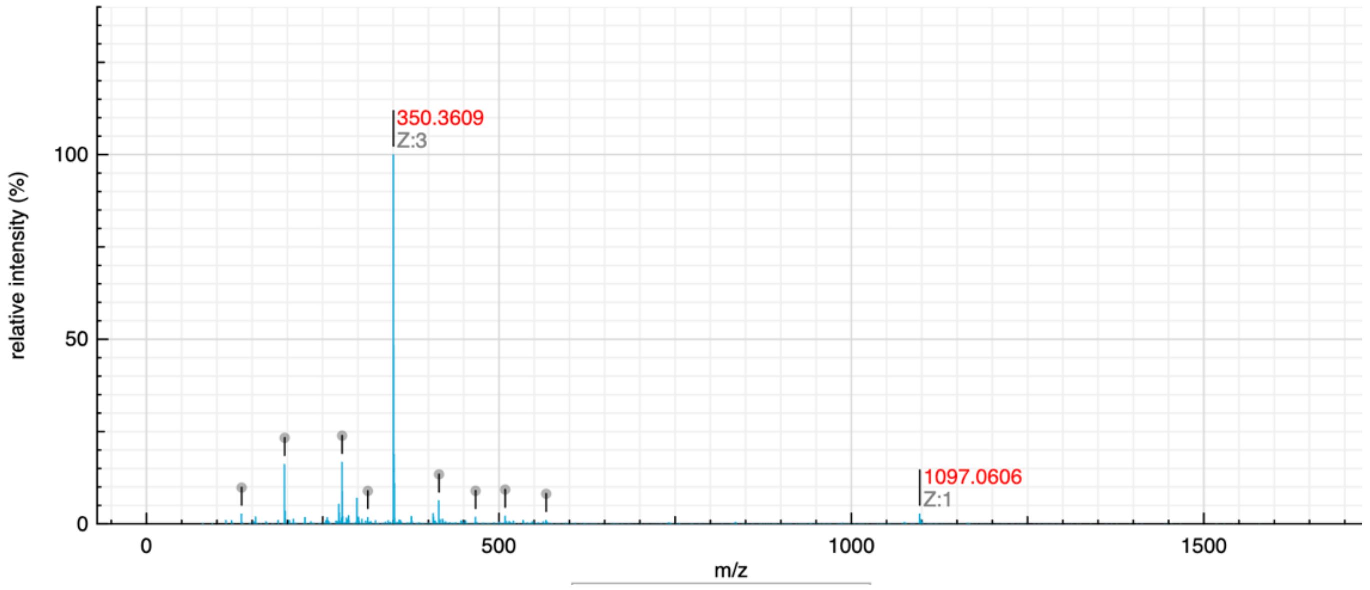

Figure S30. MS (nanochip-ESI/LTQ-orbitrap) of C4C3SO3.

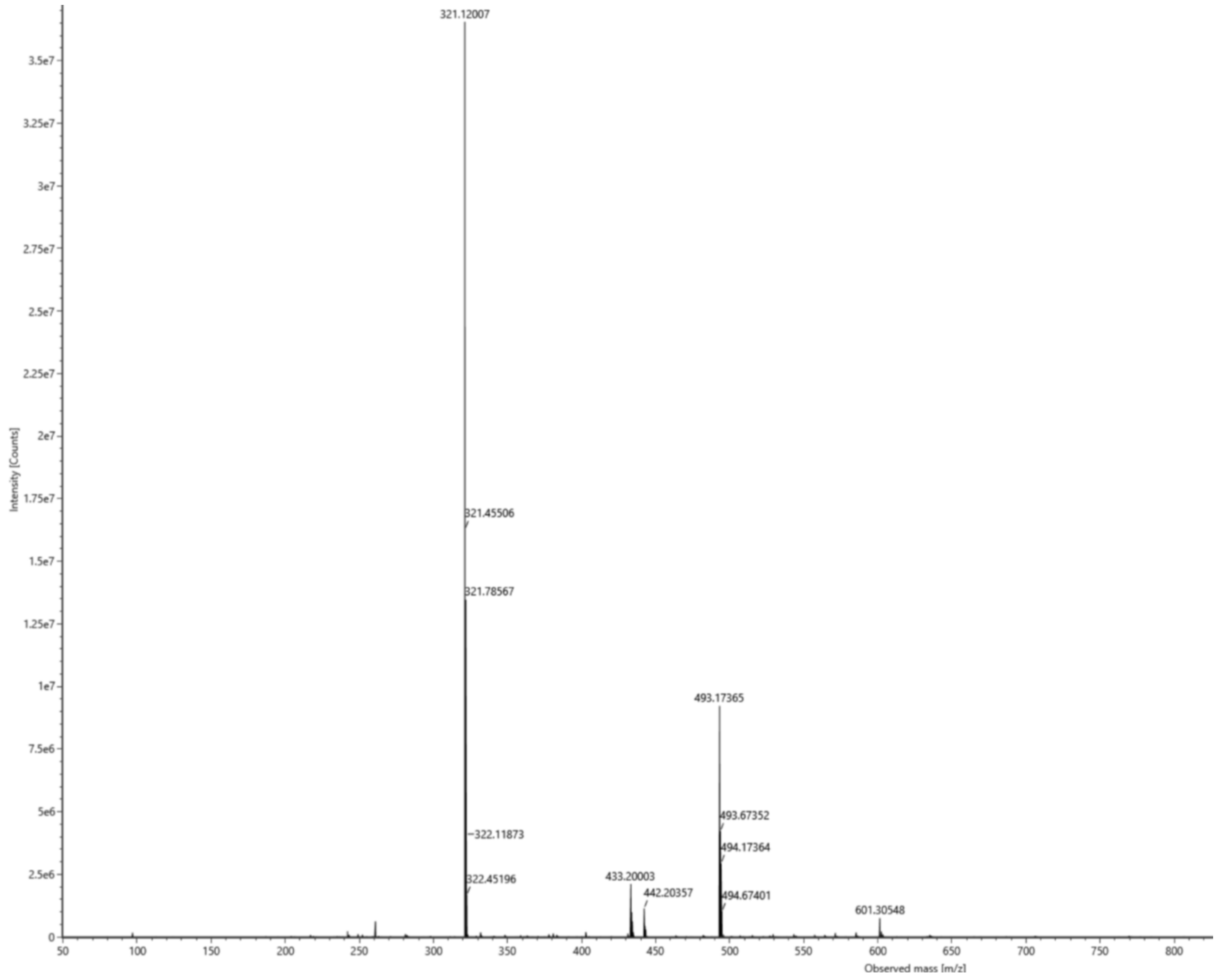

Figure S31. ESI-MS spectra of compound B3C11SO4.

References

(1) Kausar, S.; Said Khan, F.; Ishaq Mujeeb Ur Rehman, M.; Akram, M.; Riaz, M.; Rasool, G.; Hamid Khan, A.; Saleem, I.; Shamim, S.; Malik, A. A Review: Mechanism of Action of Antiviral Drugs. Int J Immunopathol Pharmacol 2021, 35, 20587384211002621. https://doi.org/10.1177/20587384211002621.

(2) Zhu, Y.; Gasbarri, M.; Zebret, S.; Pawar, S.; Mathez, G.; Diderich, J.; Valencia-Camargo, A. D.; Russenberger, D.; Wang, H.; Silva, P. H. J.; Dela Cruz, J. B.; Wei, L.; Cagno, V.; Münz, C.; Speck, R. F.; Desmecht, D.; Stellacci, F. Benzene with Alkyl Chains Is a Universal Scaffold for Multivalent Virucidal Antivirals. ACS Cent. Sci. 2024, 10 (5), 1012–1021. https://doi.org/10.1021/acscentsci.4c00054.
